# Complex Involvement of the Extracellular Matrix, Immune Effect, and Lipid Metabolism in the Development of Idiopathic Pulmonary Fibrosis

**DOI:** 10.3389/fmolb.2021.800747

**Published:** 2022-01-31

**Authors:** Weiping Qian, Shu Xia, Xiaoyun Yang, Jiaying Yu, Bingpeng Guo, Zhengfang Lin, Rui Wei, Mengmeng Mao, Ziyi Zhang, Gui Zhao, Junye Bai, Qian Han, Zhongfang Wang, Qun Luo

**Affiliations:** ^1^ Department of Respiratory Medicine, The First Affiliated Hospital of Guangzhou Medical University, Guangzhou, China; ^2^ National Clinical Center for Respiratory Disease, Guangzhou Institute of Respiratory Health, Guangzhou, China; ^3^ State Key Laboratory of Respiratory Disease, The First Affiliated Hospital of Guangzhou Medical University, Guangzhou, China

**Keywords:** idiopathic pulmonary fibrosis, mRNA sequencing, extracellular matrix remodeling, lipid metabolism, immune effect

## Abstract

**Background and objective:** Idiopathic pulmonary fibrosis (IPF) is an aggressive fibrotic pulmonary disease with spatially and temporally heterogeneous alveolar lesions. There are no early diagnostic biomarkers, limiting our understanding of IPF pathogenesis.

**Methods:** Lung tissue from surgical lung biopsy of patients with early-stage IPF (*n* = 7), transplant-stage IPF (*n* = 2), and healthy controls (*n* = 6) were subjected to mRNA sequencing and verified by real-time quantitative PCR (RT-qPCR), immunohistochemistry, Western blot, and single-cell RNA sequencing (scRNA-Seq).

**Results:** Three hundred eighty differentially expressed transcripts (DETs) were identified in IPF that were principally involved in extracellular matrix (ECM) remodeling, lipid metabolism, and immune effect. Of these DETs, 21 (DMD, MMP7, POSTN, ECM2, MMP13, FASN, FADS1, SDR16C5, ACAT2, ACSL1, CYP1A1, UGT1A6, CXCL13, CXCL5, CXCL14, IL5RA, TNFRSF19, CSF3R, S100A9, S100A8, and S100A12) were selected and verified by RT-qPCR. Differences in DMD, FASN, and MMP7 were also confirmed at a protein level. Analysis of scRNA-Seq was used to trace their cellular origin to determine which lung cells regulated them. The principal cell sources of DMD were ciliated cells, alveolar type I/II epithelial cells (AT cells), club cells, and alveolar macrophages (AMs); MMP7 derives from AT cells, club cells, and AMs, while FASN originates from AT cells, ciliated cells, and AMs.

**Conclusion:** Our data revealed a comprehensive transcriptional mRNA profile of IPF and demonstrated that ECM remodeling, lipid metabolism, and immune effect were collaboratively involved in the early development of IPF.

## Introduction

Idiopathic pulmonary fibrosis (IPF) is a chronic, progressive, fibrosing interstitial lung disease (ILD) of unknown cause, characterized by a histopathologic and/or radiologic pattern of usual interstitial pneumonia (UIP) with spatial heterogeneity, temporal heterogeneity, and honeycomb lesions (Lederer and Martinez, 2018; Lynch et al., 2018; Plantier et al., 2018; Raghu et al., 2018). IPF is difficult to differentiate from other ILDs (Schoenheit et al., 2011). Effective treatment is largely confined to lung transplantation, but this is limited by donor availability and transplantation complications (Mushiroda et al., 2008; Lederer and Martinez, 2018; Raghu et al., 2018). IPF treatments in the clinic have consistently failed, in part due to the limited understanding of IPF and lack of predictive diagnostic/prognostic biomarkers. Over the past decades, many studies (Martinez et al., 2017; Desai et al., 2018; Morse et al., 2019; Sivakumar et al., 2019) have revealed that IPF is associated with extracellular matrix (ECM) remodeling, lipid metabolism, and immune effect. For example, repeat exposure to a damaging environment can cause alveolar epithelial damage, while alveolar type II epithelial cells (AT2) are abnormally activated to initiate pulmonary fibrosis and promote proliferation, apoptosis, aging, and partial epithelial–mesenchymal transition (Martinez et al., 2017). In addition, Morse et al. reported that macrophages are highly plastic and functionally heterogeneous in IPF (Morse et al., 2019). Patients with IPF also show downregulation of cholesterol homeostasis pathway-related genes (Sivakumar et al., 2019) and low peripheral blood HDL-C levels (Aihara et al., 2013). Although these studies have shed light on pathways that could lead to IPF, our understanding of their involvement remains very limited. Several studies have utilized mRNA-Seq (Konishi et al., 2009; Steele et al., 2015; Kusko et al., 2016; Paplińska-Goryca et al., 2019; Wang et al., 2019) to identify genes and/or pathways that are differentially regulated, providing clues to specific mechanisms that underlie the occurrence and development of IPF. Nonetheless, these have been based on transplant-stage IPF samples. Because of the heterogeneity of UIP in IPF, a broader array of IPF lung tissue (including early and transplant stage) would provide more complete biological information about the IPF transcriptome and more clues to the common pathogenesis of IPF.

In the present study, we hypothesized that mRNA-Seq analysis of IPF lung tissue obtained from surgical lung biopsy (SLB) and lung transplantation would provide more comprehensive biological information and facilitate early diagnosis and a better understanding of the comprehensive pathogenesis of heterogeneous IPF. We performed mRNA-Seq on lung tissue from a cohort of patients with early-stage IPF (*n* = 7) who underwent SLB and those with transplant-stage IPF (*n* = 2) who underwent lung transplantation and compared the results with tissue obtained from healthy controls. Of the 380 differentially expressed transcripts (DETs), 21 target DETs that were mainly related to ECM remodeling, immune effect, and lung lipid metabolism-related pathways were selected for further verification by real-time quantitative PCR (RT-qPCR) and single-cell RNA sequencing (scRNA-Seq) analysis. Our findings highlighted the central and coordinatin*g* roles of different cells, pathways, and genes in the development of IPF.

## Materials and Methods

### Study Design and Inclusion Criteria for Participants

This was a retrospective, single‐center study that involved patients with IPF and healthy control (HC) treated at the ILD Center of the First Affiliated Hospital of Guangzhou Medical University between 2014 and 2018. The study protocol was approved by the local independent ethics committee (2020-71). Informed consent was obtained from participants.

### mRNA Expression Analysis

Total RNAs of tissue were extracted using a Trizol reagent kit, and sequencing and analysis performed using the Illumina novaseq 6000 platform and Bowtie2, HISAT2 (v2.1.0), StringTie (v1.3.1), and R package gmodels, respectively. Patient inclusion and exclusion criteria, methods of mRNA expression analysis, 21 target gene selection and RT-qPCR, protein expression, and cell sources of DMD, MMP7, and FASN are detailed in the Supplementary Materials.

### Statistical Analysis

Total transcript expression data were analyzed using Fisher's exact test or Wald test at a false discovery rate (FDR) of 5% and |log2FC| > 1. Statistical analysis for RT-qPCR and Western blot (WB) was performed with a two-tailed Student's t-test using GraphPad Prism software (v 8.0.2.263), or SPSS (v25.0). Results are shown as mean ± SD in the table or mean ± SEM in figures. All expression experiments conducted *in vitro* were repeated at least three times with three samples. Statistical significance was set at *p* < 0.05.

## Results

### Clinical Characteristics of Participants

**FIGURE 1:**
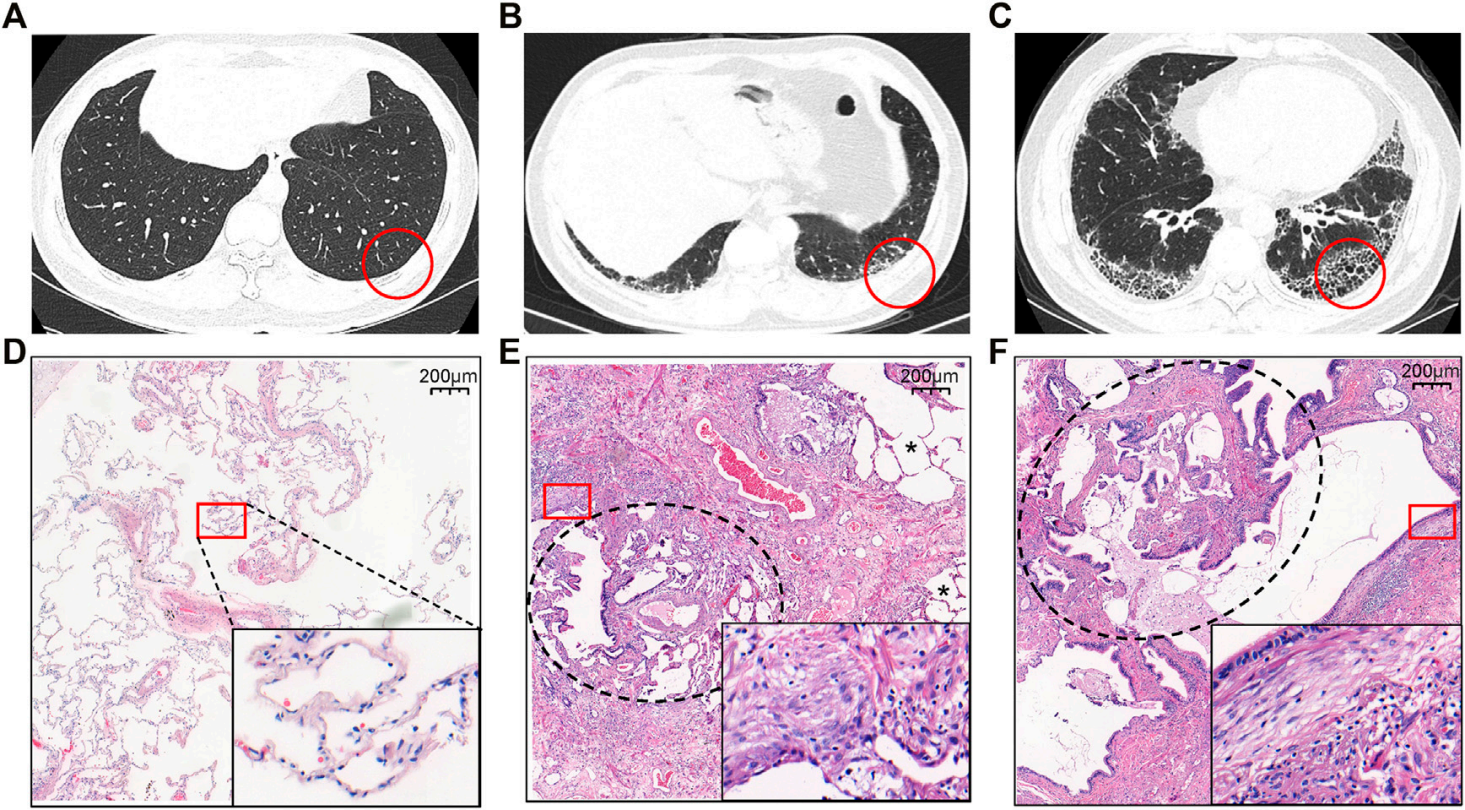
Representative HRCT **(A–C)** and histology **(D–F)** findings in patients with IPF **(B, C, E, F)** and healthy controls **(A, D)**. **(A and D)** The normal appearance of HRCT and histopathology from HC. **(B)** The HRCT topographies in the IPF patients at early stage of SLB demonstrating subpleural, basal-predominant (often heterogeneous) subtle reticulation, mild ground glass opacity (GGO), or distortion (indeterminate for UIP). **(C)** The HRCT topographies in the IPF patients at the transplant stage from lung transplantation demonstrating a typical UIP with characteristics of subpleural, basal-predominant (heterogeneous) honeycombing, and bronchiolectasis. **(E and F)** Histopathology patterns of UIP, characterized by dense fibrosis with a predilection for subpleural and paraseptal parenchyma with associated architectural distortion in the form of microscopic honeycomb change black circle pointed juxtaposed with relatively unaffected lung parenchyma (*) and fibroblast foci (square circle). Among them, E comes from the IPF lung tissue of SLB, with more relatively unaffected lung parenchyma (*), while F is from transplanted IPF lung tissue, with honeycomb change (black circle). The red circle is the area of the biopsy shown in HRCT, while the square circle is used for magnification to present very dense fibrosis (collagen fibers and fibroblasts). Abbreviation: SLB, surgical lung biopsy.

Ten patients with suspected IPF and six healthy controls were initially recruited to the study. IPF was confirmed in nine out of the ten patients (seven with early-stage disease and two with transplant-stage disease, all with a pathological UIP pattern). Compared to healthy lung tissue sections (Figures 1A, D), patients with early-stage IPF had fewer fibrotic lesions [shown in high-resolution computed tomography (HRCT) (Figure 1B)], retained more alveolar units (shown by hematoxylin and eosin staining) (Figure 1E) and had better lung function than those with transplant-stage IPF which is characterised by typical UIP along with significant honeycomb change (Figures 1C, F). Characteristics and comparison of both groups are shown in Table 1, Supplementary Table S1, Figure 1, and Supplementary Figure S2.

**TABLE 1:** Characteristics of participants in mRNA-Seq study.

	IPF (*n* = 9)	HC (*n* = 6)	*t* value	*p*-value
Age, year	57.67 ± 8.34	33.33 ± 10.50	5.00	0.0044
Male gender, No. (%)	9 (100)	6 (100)	NA	NA
Race (No.)	Asian (9)	Asian (6)	NA	NA
Lung sample source	9	6	NA	NA
SLB	7	0	NA	NA
Transplantation	2	6	NA	NA
FVC pred, %	79.69 ± 26.38	NA	NA	NA
DLco pred, %	58.75 ± 22.57	NA	NA	NA

Data are presented as mean ± SD. The *p*‐value was obtained using a two-tailed Student’s t-test. Abbreviations: SLB, surgical lung biopsy; NA, not available; FVC, forced vital capacity; DLco, diffusing capacity of lungs for carbon monoxide.

### IPF mRNA-Seq Data

**FIGURE 2:**
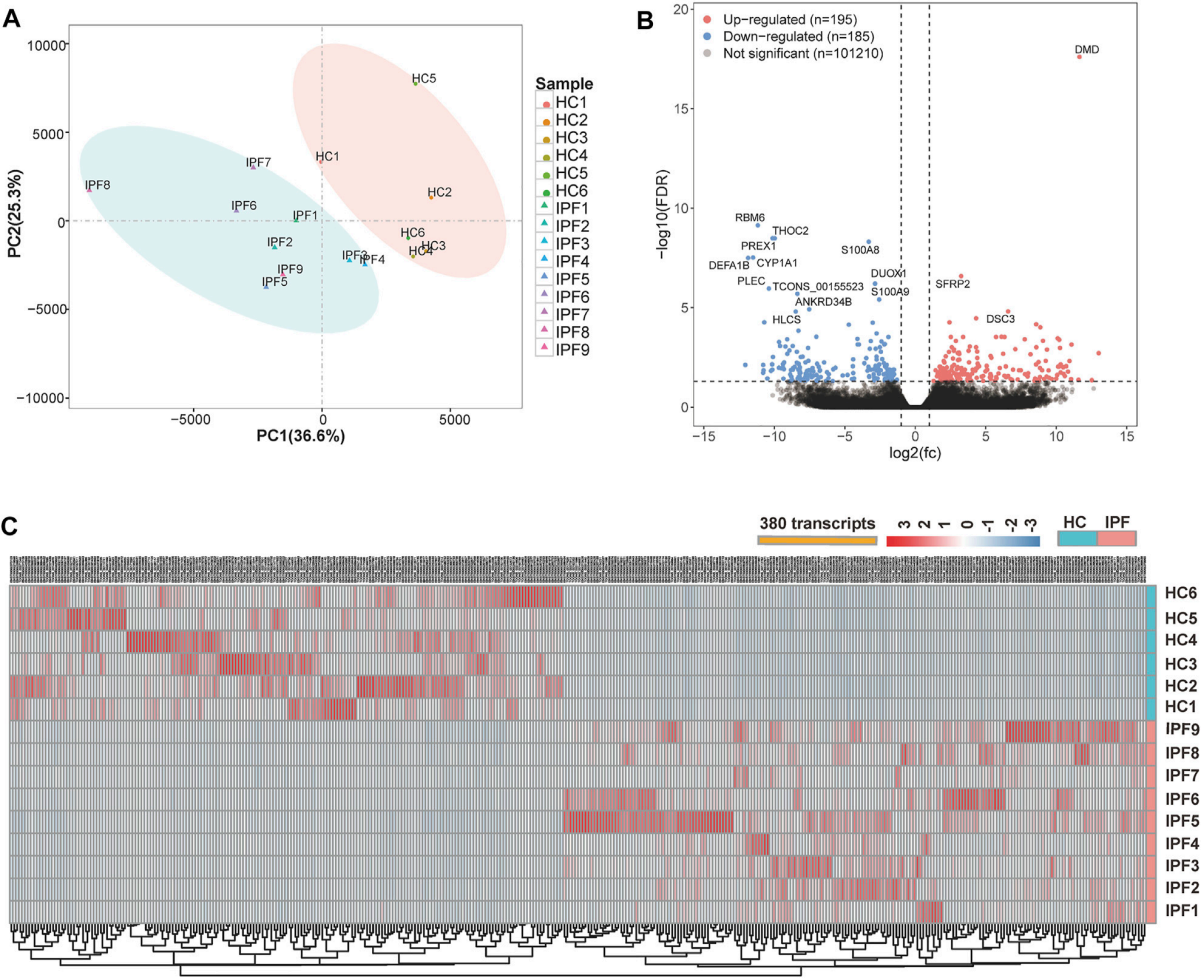
Overview of the differential expression of transcripts between lung tissue samples from patients with IPF and healthy controls. **(A)** Principal component analysis of the mRNA-Seq for lung tissue samples shows a clear separation of the samples from HC and samples from patients with IPF. **(B)** Volcano plots depict significantly dysregulated gene changes of the IPF versus control at a false discovery rate (FDR) ＜0.05 and |log2FC| > 1. Downregulated genes are shown in blue, and upregulated genes shown in red. DMD is a transcript with the lowest *p*-value. **(C)** Heat map shows the overall distribution of each differentially expressed transcript in all samples from IPF and HC. Abbreviations: FC, fold change; IPF1–IPF7, the lung samples of IPF with early stage from SLB; IPF8–IPF9, the lung samples of IPF with transplant stage from lung transplantation.

The mRNA-Seq experiments were performed using nine IPF and six HC lung tissue samples, and 101,590 transcripts were identified. Principal component analysis of all transcripts revealed that the transcriptional mRNAs of IPF tissue differed to those of the HC samples (Figure 2A). A total of 380 DETs in the IPF group were identified at an FDR of 5% and |log2FC| > 1, of which 195 were upregulated and 185 were downregulated. Interestingly, there were some significantly different DETs, including DMD, RBM6, S100A8, S100A9, and CYP1A1 (Figure 2B and Supplementary Table S2). Figure 2C shows the heat map of 380 DETs cluster analysis.

### Gene Ontology and Kyoto Encyclopedia of Genes and Genomes Pathway Enrichment Analyses

**FIGURE 3:**
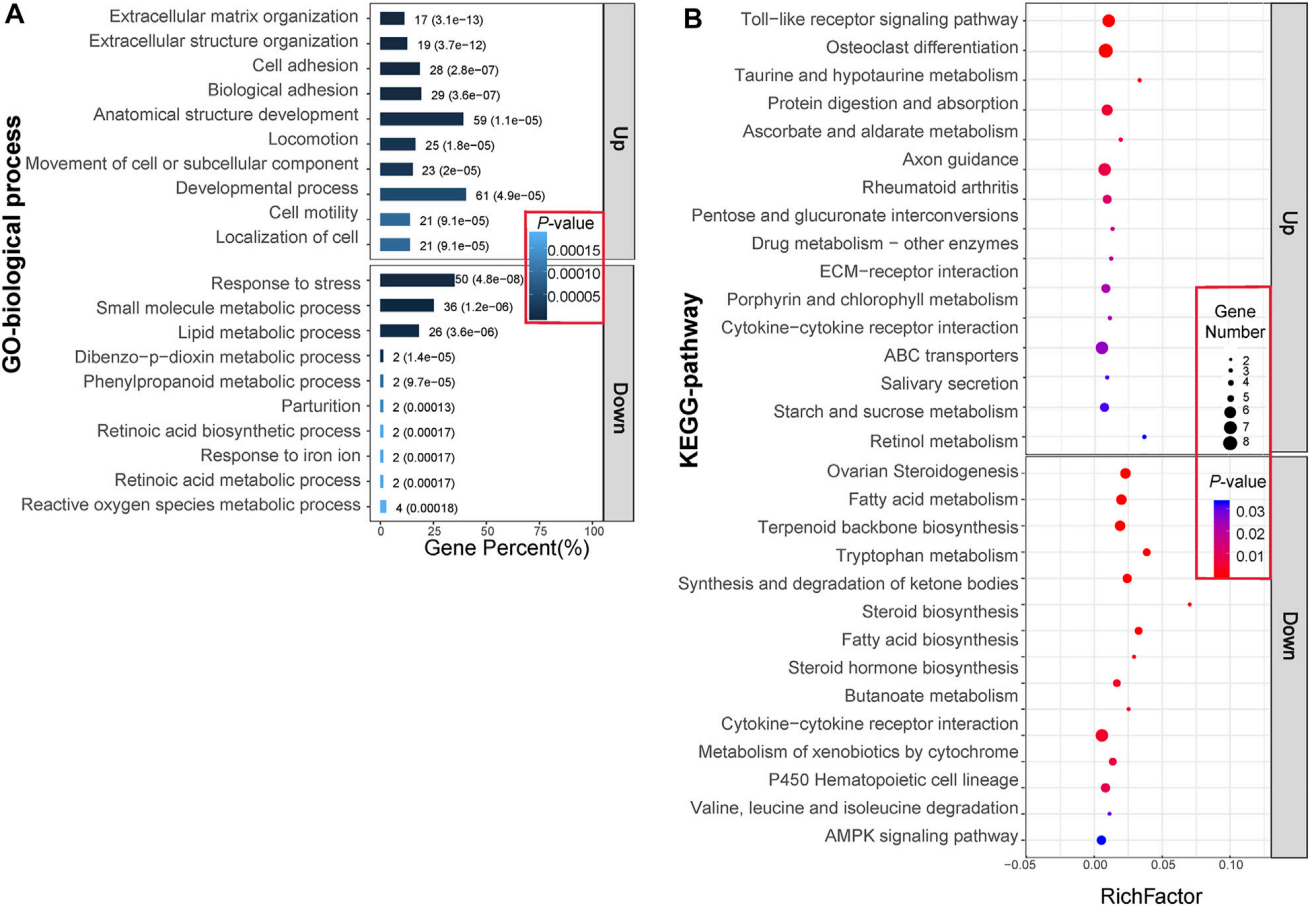
GO **(A)** and KEGG **(B)** enrichment analysis of 380 differentially expressed transcripts (DETs). **(A)** The bar graphs represent the top 10 BP terms with the lowest *p*-value for upregulated and downregulated gene expression patterns. The numbers and parenthesis value on each bar graph separately represent the numbers of DETs enriched in it and the *p*-value. **(B)** The solid dots represent the top 15 KEGG pathways with the lowest *p*-values for upregulated and downregulated gene expression patterns. Different colored dots indicate the different *p*-value, while dots of different diameters represent the numbers of DETs enriched. Abbreviations: Up, upregulation in IPF group; Down, downregulation in IPF group.

Following Gene Ontology (GO) and Kyoto Encyclopedia of Genes and Genomes (KEGG) pathway analyses for downregulated and upregulated DETs, significant BP (biological process) terms of GO and KEGG pathways were identified and are shown in Figure 3. Processes enriched by upregulated DETs involved principally ECM organization, extracellular structure organization, and cell adhesion. Those enriched BPs were largely related to response to stress, small molecule metabolic processes, and lipid metabolic processes. The KEGG pathways enriched by upregulated DETs were mainly the Toll-like receptor signaling pathway and ECM–receptor interaction, while KEGG pathways enriched by downregulated DETs involved fatty acid metabolism and fatty acid biosynthesis. Interestingly, the cytokine–cytokine receptor interaction pathway was enriched by both upregulated and downregulated DETs. Taken together, these enriched pathways were classified into three main biological processes: ECM remodeling, lipid metabolism, and immune effect.

### Selection and Verification of Target Genes

**FIGURE 4:**
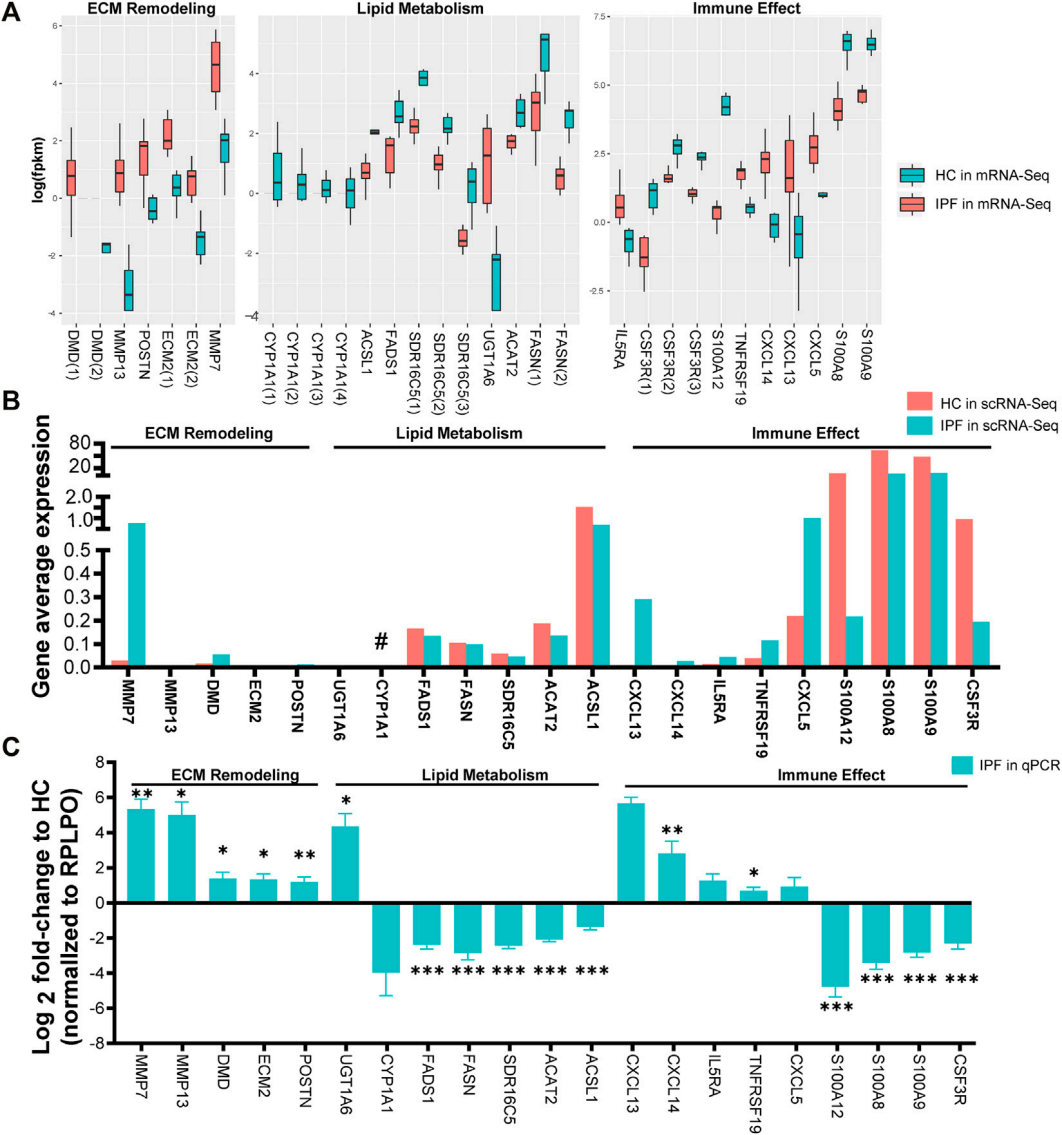
Selection and verification of 21 target genes related to ECM remodeling, lipid metabolism, and immune effect. **(A)** The fpkm expression of 21 target genes related to ECM remodeling, lipid metabolism, and immune effect via mRNA-Seq analysis from lung tissues of IPF and HC. fpkm, fragments per kilobase million; Gene(NO.) represents having one or more differential transcripts of the gene in IPF compared with HC. **(B)** The fpkm expression of the 21 target genes via scRNA-Seq data from lung tissue of 9 (6 from SLB, 3 from lung transplantation) IPF and 4 HC. Compared with HC, the trend of IPF gene fpkm expression detected by scRNA-Seq was opposite to that of mRNA-Seq (#). **(C)** Relative expression of the 21 target genes in lung tissue of 11 (5 from SLB, 6 from lung transplantation) IPF and 7 HC via RT-qPCR experience. RPLPO was the internal reference. The bar graphs show mean ± SEM. Statistical significance was set at *p* < 0.05, and *p < 0.05, **p < 0.01, and ***p < 0.001 compared with HC group using a two-tailed Student's t-test.

Target genes were selected based on the criteria of |log2FC| > 2 and genes expressed in at least two-thirds of IPF or HC samples. In total, 21 target genes were selected and classified into the following groups: 1) DMD, MMP7, POSTN, ECM2, and MMP13 related to ECM remodeling, 2) FASN, FADS1, SDR16C5, ACAT2, ACSL1, CYP1A1, and UGT1A6 associated with lipid metabolism, and 3) CXCL13, CXCL5, CXCL14, IL5RA, TNFRSF19, CSF3R, S100A9, S100A8, and S100A12 allied with immune effect. Following mRNA-Seq analysis, we determined that each of the 21 target genes may have multiple DETs: two transcripts in DMD, two in ECM, four in CYP1A1, three in SDR16C5, two in FASN, and three in CSF3R (Figure 4A). In addition, analysis of IPF scRNA-Seq data (unpublished) with deeper sequencing revealed that, except for CYPA1A1, the trend of the relative expression in IPF over HC was similar to that of mRNA-Seq (Figure 4B). RT-qPCR results also revealed that 11 genes (DMD, MMP7, POSTN, ECM2, MMP13, UGT1A6, CXCL13, CXCL5, CXCL14, IL5RA, and TNFRSF19) were upregulated, while 10 genes (FASN, FADS1, SDR16C5, ACAT2, ACSL1, CYP1A1, CSF3R, S100A9, S100A8, and S100A12) were downregulated when compared with HC (Figure 4C).

### Further Analyses of DMD, MMP7, and FASN

**FIGURE 5:**
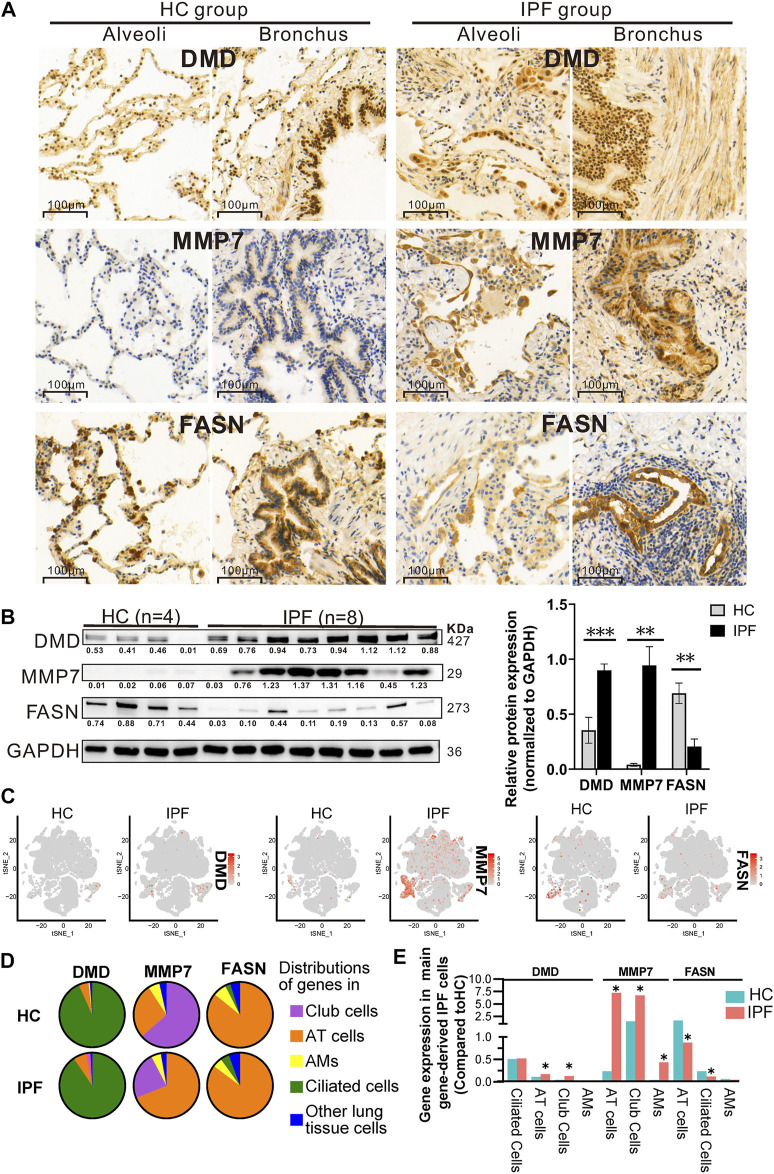
Protein expression of and cell origins of DMD, MMP7, and FASN based on multiple analyses of IHC **(A)**, WB **(B)**, and scRNA-Seq **(C–E)**. **(A)** The protein distribution of DMD, MMP7, and FASN in lung tissue of IPF and HC via IHC experience. Each gene displays protein expression in the two fields of alveoli and bronchi. In addition, brown indicates that the gene has protein expression, and the darker the color, the greater the expression. **(B)** The protein expression of DMD, MMP7, and FASN in lung tissue of IPF and HC by WB experience, showing WB exposure bands (on the left) and bar charts of statistical analysis (on the right). Lung tissue derived from 8 IPF (4 from SLB and 4 from transplantation) and 4 HC. And GAPDH served as the internal reference. Error bars indicate mean ± SEM, and their three results were consistent with that of mRNA-Seq according to a two-tailed Student's t-test. *p < 0.05, **p < 0.01, ***p < 0.001. **(C–E)** tSNE plots **(C)** displaying the expression level of DMD, MMP7, and FASN in all cell clusters from IPF and HC, pie chart **(D)** showing three lung cell sources expressing DMD, MMP7, and FASN genes, and histogram **(E)** showing the differential expression of the first three cells of DMD, MMP7, and FASN in IPF and HC groups. Compared with HC, the gene expression of DMD, MMP7, and FASN in IPF was upregulated or downregulated by at least 1.5-fold (*).

To further verify the regulated genes, WB analysis was performed using IPF and HC tissue for DMD (the most differentially expressed transcript in mRNA-Seq analysis), MMP7 and FASN (the most significant fold change and significant statistical change in RT-qPCR). The immunohistology chemistry (IHC) results in Figure 5A show that: 1) DMD protein was expressed in the majority of the lung tissues and lung cells, mainly in the nucleus around the alveoli and around the bronchial lumen but also in the cytoplasm and in the muscle cell membrane in the IPF and HC groups; 2) MMP7 protein was clearly evident in the cytoplasm around the alveoli and bronchial lumen in the IPF group, but expression was low in the HC group; and 3) protein distribution of FASN was similar to that of DMD, mainly in the cytoplasm around the alveoli and bronchial lumen, and the staining of HC was darker than that in IPF. Next, we performed WB and further conformed that the protein expression of DMD and MMP7 in the IPF group was also significantly higher than that in the HC group, while the protein expression of FASN was clearly lower (Figure 5B). Finally, using the IPF scRNA-Seq data, we compared the cell sources of differentially expressed DMD, MMP7, and FASN genes in IPF and HC (Figures 5C, D). We demonstrated that DMD was mainly expressed in ciliated cells, AT cells, and alveolar macrophages (AMs) in HC while mostly expressed in ciliated cells, AT cells, and club cells in IPF lung tissue and that DMD expression was upregulated in AT (1.5-fold) and club cells (2.7-fold) in IPF lung tissue (Figure 5E). Moreover, MMP7 was mainly expressed in AT cells, club cells, and AMs in both IPF and HC lung tissue, and it was 30.7-, 4.4-, and 11.1-fold upregulated in AT cells, club cells, and AMs of IPF. Finally, FASN was mainly expressed in AT cells, ciliated cells, and AMs in both groups and was about 2-fold downregulated in AT and ciliated cells in IPF. In addition, it is worthy to note that DMD, MMP7, and FASN were higher in early-stage than in the transplant-stage IPF tissue (Supplementary Figure S1).

## Discussion

IPF is usually characterized by a histopathologic and/or radiologic pattern of UIP with spatial heterogeneity, temporal heterogeneity, and the presence of honeycomb lesions (Lederer and Martinez, 2018; Lynch et al., 2018; Plantier et al., 2018; Raghu et al., 2018). Investigation of more comprehensive IPF specimens will help better understand the pathogenesis of IPF and unravel an in-depth mechanism underneath. Recently, an increasing number of studies have focused on the pathogenesis of IPF using high-throughput mRNA-Seq and scRNA-Seq. Nonetheless, the sources of tissue of the most previous IPF transcriptome studies are mainly from patients undergoing lung transplantation (Vukmirovic et al., 2017; Luzina et al., 2018; McDonough et al., 2019), which is generally considered as “late” IPF tissue. In this study, in addition to lung tissue specimens from transplant-stage IPF, more early-stage IPF specimens obtained from SLB were included for mRNA-Seq analysis. This is helpful to identify the complete transcriptional profile of IPF and help better the understanding of earlier development of IPF. In the present study, we identified several important pathways and categorized them into three biological processes, including ECM remodeling, lipid metabolism, and immune effect, and genes (DMD, MMP7, POSTN, ECM2, MMP13, FASN, FADS1, SDR16C5, ACAT2, ACSL1, CYP1A1, UGT1A6, CXCL13, CXCL5, CXCL14, IL5RA, TNFRSF19, CSF3R, S100A9, S100A8, and S100A12) involved in the processes. Additionally, we demonstrated the differential expression of DMD, MMP7, and FASN in AT cells, AMs, club cells, and ciliated cells of IPF.

Several genes such as MMP7, CSF3R, and S100A12 identified and characterized in our study have previously been reported (Deng et al., 2018; Chung et al., 2019; McDonough et al., 2019). For instance, we demonstrated that MMP7 was mainly enriched in ECM organization and ECM–receptor interaction, similar to a previous study, which described that MMP7 in blood levels has been consistently associated with IPF disease progression and survival (McDonough et al., 2019). We found that MMP7 mainly upregulated in the bronchi and alveoli of IPF; in addition, the main cell sources of MMP7 were AT cells, club cells, and AMs based on scRNA-Seq analysis. FASN was mainly enriched in fatty acid synthase and mainly downregulated in AT cells, ciliated cells, and AMs in the lung tissues of IPF. In fact, the phenomenon of lipid metabolism disorder in IPF patients has also been verified in animal models of fibrosis; for example, Chung et al. (2019) found the loss of AEC2 cell-specific FASN in bleomycin-induced pulmonary fibrosis in mice and the loss of lipid synthesis in AEC2 cells during mitochondrial injury could aggravate pulmonary fibrosis.

Despite the confirmation of inflammatory cytokines and innate and adaptive immune cell infiltration in IPF, several clinical experiments of immunosuppressive therapy for IPF have failed. This has downplayed the role of chronic inflammation and immune effect in the pathogenesis of IPF (Thannickal et al., 2014). Nonetheless, according to our current research results, failure of therapies based on immune intervention may be due to limited knowledge about the involvement of immune disorders in IPF. In our study, a great number of immune factors were altered in IPF patients, including upregulation of CXCL5, CXCL13, CXCL14, IL5RA, and TNFRSF19 and downregulation of CSF3R, S100A8, S100A9, and S100A12. Leurs and Lindholm (2013) reported that S100A12 was involved in the activation of signal transduction pathways in endothelial cells, vascular smooth muscle cells, and inflammatory cells, further leading to the transcription and secretion of proinflammatory cytokines and cell adhesion molecules. It has been reported that a high concentration of S100A12 in peripheral blood indicated a low overall survival rate of IPF, suggesting that the excessive inflammatory-immune response led to a worse prognosis (Richards et al., 2012). In addition to immune-related genes, there are also reports about immune cells in relation to IPF. Duffield et al. (2005) and Wynn (2011) revealed AMs were depleted in the early inflammation/maintenance phase of the fibrotic reaction in CD11b-DTR mice. This may have led to a relative reduction in scar formation and myofibroblasts, although fibrosis continued if AMs were consumed in the late remodeling/recovery stage of fibrosis.

DMD is a novel gene closely related to ECM remodeling in IPF. We found that DMD was mainly expressed by ciliated cells and secondarily expressed in AT and club cells in the lung tissue. Interestingly, there was an about 1.5-fold upregulation of DMD in AT and club cells of IPF tissue, while there was no significant difference between the ciliated cells of IPF and HC groups, suggesting that the role of DMD in IPF development may be cell type-dependent. It is worthy of note that the DMD gene is one of the largest protein-coding genes in the human genome, existing on the X chromosome, and encodes and synthesizes 427 KDa dystrophin protein that is distributed principally in the myocardium, smooth muscle, and skeletal muscle (Tuffery-Giraud et al., 2017). Edematous cells in Duchenne and Becker muscular dystrophy, X-linked dilated cardiomyopathy, and other muscle diseases result in cell fragmentation, leading to increased intracellular calcium ions that serve as a second messenger to activate the inflammatory cascade in organs (Sironi et al., 2002; Nardes et al., 2012; Kamdar and Garry, 2016). At present, the mainstream theory about the muscle degeneration and fibrosis causes of Duchenne muscular dystrophy (Mann et al., 2011) is that the integrity of the dystrophin protein in muscle cell membrane is lost and destroyed. When mechanically stimulated, sodium and calcium influx can be amplified and promote inflammatory cells to release various fibrogenic factors with consequent proliferation and differentiation of fibroblasts into myofibroblasts. Both can secrete ECM and ECM remodeling factors, with a subsequent imbalance of ECM synthesis and degradation and with consequent fibrosis. The histological feature of IPF is the gradual deposition of ECM and the gradual decrease in parenchymal cells. We hypothesize that dystrophin protein lesions occur in the lung tissue of IPF, especially in AT and club cells, and may lead to an imbalance between ECM synthesis and degradation in the lungs, promoting the occurrence and development of IPF.

The results of our study, from changes in the overall molecular level of IPF to alterations in the lung cells, showed that ECM remodeling, lipid metabolism, and immune effect are complex during the early development of IPF, suggesting that no single cell, gene, or pathway can contribute to the complex and heterogeneous nature of the disease. In addition, our findings supplement and reinforce the present transcriptome profile of human IPF disease.

## Data Availability

The datasets presented in this study can be found in online repositories. The names of the repository/repositories and accession number(s) can be found below: NGDC Genome Sequence Archive, accession no: HRA001639.

## Abbreviations

ACAT2, acetyl-CoA acetyltransferase 2; ACSL1, acyl-CoA synthetase long chain family member 1; CSF3R, colony stimulating factor 3 receptor; CXCL13, C-X-C motif chemokine ligand 13; CXCL14, C-X-C motif chemokine ligand 14; CXCL5, C-X-C motif chemokine ligand 5; CYP1A1, cytochrome P450 family 1 subfamily A member 1; DMD, dystrophin; ECM2, extracellular matrix protein 2; FADS1, fatty acid desaturase 1; FASN, fatty acid synthase; IL5RA, interleukin 5 receptor subunit alpha; MMP13, matrix metallopeptidase 13; MMP7, matrix metallopeptidase 7; POSTN, periostin; S100A12, S100 calcium binding protein A12; S100A8, S100 calcium binding protein A8; S100A9, S100 calcium binding protein A9; SDR16C5, short chain dehydrogenase/reductase family 16C member 5; TNFRSF19, tumor necrosis factor receptor superfamily member 19; UGT1A6, UDP glucuronosyltransferase family 1 member A6; SLB, surgical lung biopsy; IPF, idiopathic pulmonary fibrosis; HC, healthy controls.
