# Supplementary material for: Complex Involvement of the Extracellular Matrix, Immune Effect, and Lipid Metabolism in the Development of Idiopathic Pulmonary Fibrosis
Source: Front Mol Biosci. 2022 Jan 31;8:800747. doi: 10.3389/fmolb.2021.800747 (PMC8841329; doi:10.3389/fmolb.2021.800747)
Supplement: Supplementary file 1 [file DataSheet2.PDF]

Complex involvement of the extracellular matrix, immune effect, and lipid metabolism in the development of idiopathic pulmonary fibrosis

Weiping Qian[1,3]*, Shu Xia[1,3]*, Xiaoyun Yang[2,3]*, Jiaying Yu[2,3], Bingpeng Guo[1,3], Zhengfang Lin[2,3] Rui Wei[1,3],Mengmeng Mao[1,3], Ziyi Zhang[1,3], Gui Zhao[1,3], Junye Bai[1,3], Qian Han[1,3#], Zhongfang Wang[2,3#], Qun Luo[1,3#]

[1] Department of Respiratory Medicine, The First Affiliated Hospital of Guangzhou Medical University, Guangzhou, Guangdong, P.R. China

[2] State Key Laboratory of Respiratory Disease, The First Affiliated Hospital of Guangzhou Medical University, Guangzhou, Guangdong, P.R. China

[3] National Clinical Center for Respiratory Disease, Guangzhou Institute of Respiratory Health, Guangzhou, Guangdong, P.R. China

*Corresponding author: Prof. Qun Luo or Prof. Zhongfang Wang or Prof. Qian Han

State Key Laboratory of Respiratory Disease, National Clinical Center for Respiratory Disease, Guangzhou Institute of Respiratory Health, The First Affiliated Hospital of Guangzhou Medical University. Address for correspondence:151 Yanjiang West Road, Guangzhou, CHINA. Email: luoqunx@163.com or wangzhongfang@gird.cn or hanqian1020@yahoo.com. Phone number:020-83062859.

QWP*, XS*, and YXY* contributed equally to this article,

LQ#, WZF#, and HQ# contributed equally to this article

ONLINE DATA SUPPLEMENT

The inclusion and exclusion criteria of mRNA-Seq

The inclusion criteria for IPF group were concordant with the official ATS/ERS/JRS/ALAT clinical practice guidelines[1, 2] and included bilateral basal crackles with or without HRCT pattern of UIP, and a multidisciplinary discussion. Sufficient lung tissues were obtained and collected by SLB or lung transplantation. For HC, the evaluation of normal lung tissue were performed by two experienced thoracic pathologists. All participants were stable, without acute exacerbation. And exclusion criteria for participants were current treatment of steroid, immunosuppressants, antibacterials， and antifibrotic therapy in the previous 4 weeks.

mRNA expression analysis

Lung tissue was ground under liquid nitrogen and total RNA extracted using a Trizol reagent kit (Life technologies) in accordance with the manufacturer's protocol. A Micro UV Spectrophotometer and Agilent 2100 were used to detect the concentration, purity, and integrity of RNA. The extracted RNA (total RNA $\geq$ 2 μg, concentration $\geq$ 70 ng/μL, and RIN $\geq$ 7) was used to construct the library. A DNA 1000 assay Kit (Agilent Technologies) was used for quality inspection of the library, followed by Illumina novaseq 6000 for sequencing.

Bioinformatics analysis

Raw reads were filtered using fastp[3] and reads mapped to the ribosome RNA (rRNA) database using the short read alignment tool Bowtie2[4]. The rRNA mapped reads were removed. The remaining clean reads were further used for subsequent mRNA-Seq. Then, the paired-end clean reads were compared with the reference genome using HISAT2 (v2.1.0) software[5] with "RNA-strandness RF" and other parameters set as default. The mapped reads of each sample were assembled using StringTie (v1.3.1) [6, 7] and a reference-based approach. For each transcription region, the FPKM (Fragments Per Kilobase

of transcript per Million mapped reads) value was calculated using RSEM to quantify its expression abundance and variations[8]. Principal component analysis (PCA) was performed with R package gmodels.

Identification and Enrichment analyses of DETs

RNA differential expression analysis between the two groups was performed using DESeq2[9] software. Genes with a false discovery rate (FDR) < 0.05 and |log2FC|＞1 were considered DETs. Gene Ontology (GO) enrichment analysis was performed on DETs[10] and the major public pathway-related database KEGG[11] was used to analyze the pathways that were significantly enriched in DETs.

Selection and qPCR validation of the 21 target genes

Selection: According to the following, 21 target genes were selected from 380 DETs for verification, (1) genes with 2/3 expression in the IPF or HC sapmles, (2) |log2FC|> 2, (3) genes enriched in the top 10 GO terms or KEGG pathways.

qPCR: A total of 21 target genes were used for qPCR verification, including DMD, MMP7, POSTN, ECM2, MMP13, FASN, FADS1, SDR16C5, ACAT2, ACSL1, CYP1A1, UGT1A6, CXCL13, CXCL5, CXCL14, IL5RA, TNFRSF19, CSF3R, S100A9, S100A8, and S100A12. The qPCR experiment was divided into IPF(n=11, 5 of IPF with early-stage from SLB and 6 of IPF with transplant-stage) and HC(n=7). First, the total RNAs of lung tissues were extracted by using Trizol reagent kit (Life technologies) according to the manufacturer's protocol. Then, total RNA was reverse-transcribed into cDNA using the HiScript III RT SuperMix for qPCR (+gDNA wiper) KIT from Vazyme Biotech Co., Ltd (Nanjing, China). The qPCR reaction was performed in accordance with the instructions of ChamQ Universal SYBR qPCR Master Mix (Vazyme Biotech Co., Ltd, Nanjing, China) in a 20 μL reaction for 40 cycles. RT-qPCR was performed and analyzed using CFX ConnectTM Optics Module (Bio-Rad Laboratories Inc., Singapore). RT-qPCR primers used in this

study (Aiji Biotechnology Co., Ltd, Guangzhou, China) are listed in Table S2. Gene expression was analyzed using the difference in quantification cycle (ΔCq) method. The results are expressed as fold change normalized to RPLPO average expression in HC samples.

Protein expressions and cell sources of DMD, MMP7 and FASN

Immunohistochemistry:A total of 3 important target genes were used for immunohistochemistry verification, including DMD, MMP7, and FASN. The immunohistochemistry experiment was divided into IPF(n=8, 4 from SLB, and 4 from lung transplantation) and HC(n=4), and the smaples were obtained from the Pathology Archives, the First Affiliated Hospital of Guangzhou Medical University, and all specimens met the inclusion criteria of this study. First,the glass slides were incubated at 60 °C for 60 min, soaked for 30 min in graded xylene rinses for de-paraffining, rehydrated in graded ethanol solutions, and then rinsed with water. Thereafter, pressure cooker antigen retrieval consisted of a period at 95 °C for 15 min in 10× Tris-EDTA solution (pH 9.0) (Solarbio, China) followed by a reduction to room temperature and washing with TBS. Then, the sections were treated with blocking Endogenous Peroxidase and blocking antigen in accordance with the instructions of UltraSensitiveTM SP (Rabbit) IHC Kit (MXB, Fuzhou, China). To label the first antigen, primary antibodies were diluted at optimal dilutions in Immunol Staining Primary Antibody Dilution Buffer (Beyotime Biotechnology, Shanghai, China) and incubated at 4 °C overnight. Sections were washed with TBST and then incubated with secondary antibody for 30 min in accordance with the instructions of the IHC Kit and then with the liquid DAB+ Substrate Chromogen System (cat# K3468) for 30 min at room temperature. Primary and secondary antibodies are detailed in Table S3. Finally, sections were rinsed in TBST to stop the reaction, mounted on gelatin-coated slides, air dried, dehydrated with 70–100% alcohol, cleaned with xylene, and then a cover-slip applied prior to being subjected to a digital pathology scanner (PRECICE 500B, Youna Technology Co., Ltd.,

Beijing, China) or microscopic examination.

Western blot experience: Then, the proteins of DMD, MMP7 ans FASN were detected by WB in four cases of HC lung tissues, four cases of IPF lung tissues with early-stage and four cases of IPF lung tissues with transplant-stage. Tissue samples were homogenized in RIPA buffer (Jingcai Biotechnology Co., Ltd., Xi'an, China). The supernatants were collected after centrifugation at 12,000 × g for 20 min at 4°C. Protein concentration was determined using a PIERCETM BCA protein assay kit (Thermo Fisher Scientific, Waltham, Massachusetts, USA). Individually, an equal amount of each protein extract (20 μg) was separated with a TGX Rapid gel preparation kit (Bio-Rad Laboratories Inc., California, USA) or a Genshare CFAS high PAGE protein electrophoresis gel preparation kit (Jingcai Biotechnology Co., Ltd., Xi'an, China) to detect proteins. GAPDH protein expression was used as the loading control. Subsequently, proteins were transferred onto a 0.45-μm hybridization nitrocellulose filter membrane (Millipore Ltd., Ireland) using the Trans-Blot TurboTM Transfer System (Bio-Rad Laboratories Inc., Singapore) or Genshare CFAS (Jingcai Biotechnology Co., Ltd., Xi'an, China). Then, membranes were blocked in 5% non-fat dry milk (5% NFDM) for 1 h at room temperature. Membranes were immunoblotted with primary antibody with optimal dilutions (Table S3) at 4 °C overnight and then incubated with either HRP-conjugated anti-rabbit immunoglobulin or HRP-linked anti-mouse immunoglobulin (Table S4) for 1 h at room temperature. Protein signals were detected with TanonTN ECL Chemiluminescence substrate (Yuwei Biotechnology Instrument Co., Ltd., Shanghai, China) using Tanon Chemiluminescence Gel Imaging system Tanon-5200Multi (Yuwei Biotechnology instrument Co., Ltd., Shanghai, China). Band intensity was also quantified by ImageJ software (v1.51).

Joint analysis with scRNA-Seq data: Our research team has used 4 cases of HC, 9 (6 in SLB and 3 in lung transplantation) cases of IPF for scRNA-Seq analysis (unpublished data). Using scRNA-Seq

data, we further analyzed the average expression of the 21 target genes in IPF and HC groups and compared them with each other. Next, we traced the cell sources of DMD, MMP7 and FASN in IPF and HC via scRNA-Seq data.

REFERENCES

1. Raghu G., Remy-Jardin M., Myers J.L., Richeldi L., Ryerson C.J., Lederer D.J., Behr J., Cottin V., Danoff S.K., Morell F., Flaherty K.R., Wells A., Martinez F.J., Azuma A., Bice T.J., Bouros D., Brown K.K., Collard H.R., Duggal A., Galvin L., Inoue Y., Jenkins R.G., Johkoh T., Kazerooni E.A., Kitaichi M., Knight S.L., Mansour G., Nicholson A.G., Pipavath S.N.J., Buendia-Roldan I., Selman M., Travis W.D., Walsh S., Wilson K.C., American Thoracic Society E.R.S.J.R.S., Latin American Thoracic S. Diagnosis of Idiopathic Pulmonary Fibrosis. An Official ATS/ERS/JRS/ALAT Clinical Practice Guideline. Am J Respir Crit Care Med. 2018;198(5):e44-e68.
2. Raghu G., Collard H.R., Egan J.J., Martinez F.J., Behr J., Brown K.K., Colby T.V., Cordier J.F., Flaherty K.R., Lasky J.A., Lynch D.A., Ryu J.H., Swigris J.J., Wells A.U., Ancochea J., Bouros D., Carvalho C., Costabel U., Ebina M., Hansell D.M., Johkoh T., Kim D.S., King T.E., Jr., Kondoh Y., Myers J., Muller N.L., Nicholson A.G., Richeldi L., Selman M., Dudden R.F., Griss B.S., Protzko S.L., Schunemann H.J., Fibrosis A.E.J.A.C.o.I.P. An official ATS/ERS/JRS/ALAT statement: idiopathic pulmonary fibrosis: evidence-based guidelines for diagnosis and management. Am J Respir Crit Care Med. 2011;183(6):788-824.
3. Chen S., Zhou Y., Chen Y., Gu J. fastp: an ultra-fast all-in-one FASTQ preprocessor. Bioinformatics. 2018;34(17):i884-i90.
4. Langmead B., Salzberg S.L. Fast gapped-read alignment with Bowtie 2. Nat Methods. 2012;9(4):357-9.
5. Kim D., Langmead B., Salzberg S.L. HISAT: a fast spliced aligner with low memory requirements. Nat Methods. 2015;12(4):357-60.
6. Pertea M., Pertea G.M., Antonescu C.M., Chang T.C., Mendell J.T., Salzberg S.L. StringTie enables improved reconstruction of a transcriptome from RNA-seq reads. Nat Biotechnol. 2015;33(3):290-5.
7. Pertea M., Kim D., Pertea G.M., Leek J.T., Salzberg S.L. Transcript-level expression analysis of RNA-seq experiments with HISAT, StringTie and Ballgown. Nat Protoc. 2016;11(9):1650-67.
8. Li B., Dewey C.N. RSEM: accurate transcript quantification from RNA-Seq data with or without a reference genome. BMC Bioinformatics. 2011;12:323.

9. Robinson M.D., McCarthy D.J., Smyth G.K. edgeR: a bioconductor package for differential expression analysis of digital gene expression data. Bioinformatics. 2010;26(1):139-40.

10. Ashburner M., Ball C.A., Blake J.A., Botstein D., Butler H., Cherry J.M., Davis A.P., Dolinski K., Dwight S.S., Eppig J.T., Harris M.A., Hill D.P., Issel-Tarver L., Kasarskis A., Lewis S., Matese J.C., Richardson J.E., Ringwald M., Rubin G.M., Sherlock G. Gene ontology: tool for the unification of biology. The Gene Ontology Consortium. Nat Genet. 2000;25(1):25-9.

11. Kanehisa M., Goto S. KEGG: kyoto encyclopedia of genes and genomes. Nucleic Acids Res. 2000;28(1):27-30.

SUPPLEMENTARY TABLES

Table S1 Specific clinical information of IPF sequencing specimens

IPF smples	Acquisition mode	Gender	Age, yr	FVC% pred	DLCO% pred
IPF1	SLB	male	63	142.4	82.3
IPF2	SLB	male	66	90.0	45.0
IPF3	SLB	male	51	85.0	87.0
IPF4	SLB	male	65	88.0	60.0
IPF5	SLB	male	57	88.1	75.3
IPF6	SLB	male	42	77.1	45.1
IPF7	SLB	male	51	53.0	56.0
IPF8	Transplantation	male	58	36.7	19.3
IPF9	Transplantation	male	66	51.1	NA

Abbreviations: NA, Not Available, becaese the IPF patient was unable to complete the diffusion function test.

Table S2.Human primer sequences for quantitative PCR experience

Primer	GenBank (NM_NO.)	Forward primer (5′-3′)	Reverse primer (5′ -3′)
DMD	004006	GCTCAACCATCGATTTGCAGCC	TTCAGCCTCCAGTGGTTCAAGC
S100A8	002964	ATGCCGTCTACAGGGATGACCT	AGAATGAGGAACTCCTGGAAGTTA
S100A9	002965	GCACCCAGACACCCTGAACCA	TGTGTCCAGGTCCTCCATGATG
S100A12	005621	CTCTAAGGGTGAGCTGAAGCAG	ACCTGTTCATCTTGATTAGCATCC
MMP7	002423	TCGGAGGAGATGCTCACTTCGA	GGATCAGAGGAATGTCCCATACC
MMP13	002427	CCTTGATGCCATTACCAGTCTCC	AAACAGCTCCGCATCAACCTGC
ECM2	001393	ATAGGATTGCTCCTTTAGCCTGG	ACTAGGTGCAGCAAGGACTTGG
POSTN	006475	CAGCAAACCACCTTCACGGATC	TTAAGGAGGCGCTGAACCATGC
CYP1A1	000499	GATTGAGCACTGTCAGGAGAAGC	ATGAGGCTCCAGGAGATAGCAG
SDR16C5	138969	TGCACGCCTATACCTGCGATTG	GGCATTGTTGATTAGGATGGAAAC
UGT1A6	001072	GCAAAGCGCATGGAGACTAAGG	GGTCCTTGTGAAGGCTGGAGAG
FASN	004104	ACAGCGGGGAATGGGTACT	GACTGGTACAACGAGCGGAT
FADS1	013402	CTGTCGGTCTTCAGCACCTCAA	CTGGGTCTTTGCGGAAGCAGTT
ACAT2	005891	TGGTGCCTTAGCTGCTGTTCCT	GGCTTGTCTAACAGGATTCTGCC
ACSL1	001995	ATCAGGCTGCTCATGGATGACC	AGTCCAAGAGCCATCGCTTCAG
CSF3R	156039	CCACTACACCATCTTCTGGACC	GGTGGATGTGATACAGACTGGC
TNFRSF19	018647	GGTGCATTCTGCAGCCAGTCTT	CAGGCATCTGAAAACTCGCCAC
CXCL13	006419	TATCCCTAGACGCTTCATTGATCG	CCATTCAGCTTGAGGGTCCACA
CXCL5	002994	CAGACCACGCAAGGAGTTCATC	TTCCTTCCCGTTCTTCAGGGAG
IL5RA	000564	TGACTGGCTTGCGGTGCTTGTT	CTGCTGTGACATTCAGTGGAGG
CXCL14	004887	AGATCCGCTACAGCGACGTGAA	GCAGTGCTCCTGACCTCGGTA
RPLPO	001002	TGGTCATCCAGCAGGTGTTCGA	ACAGACACTGGCAACATTGCGG

Table S3. The information of primary antibody and secondary antibody for IHC and WB.

Antibody	Source	Brand	Catalog	Dilutions in IHC	Dilutions in WB
MMP7	Rabbit	Abcam	ab205525	1:800	1:1000
DMD	Rabbit	Abcam	ab15277	1:50	1:500
FASN	Rabbit	CST	mAb #3180	1:50	1:500
GAPDH	Mouse	Abcepta	AM8539b-200	NA	1:2500
Anti-Mouse IgG	Goat	Jackson Immuno Research	115-035-062	NA	1:1000
Anti-Rabbit IgG	Goat	Jackson Immuno Research	111-035-045	NA	1:1000

Abbreviations: IHC, Immunohistochemistry; WB, Western Blot; NA, Not Available.

Table S4. The information of 380 DETs based on mRNA-Seq analysis of IPF

Transcript ID	Symbol	log2(FC)	*P*-value	FDR
TCONS_00203730	DMD	11.65	2.81E-23	2.49E-18
ENST00000266022	RBM6	-11.17	1.64E-14	7.26E-10
TCONS_00119741	PREX1	-10.12	1.25E-13	3.27E-09
TCONS_00205714	THOC2	-9.97	1.47E-13	3.27E-09
ENST00000368733	S100A8	-3.30	2.75E-13	4.87E-09
ENST00000395048	CYP1A1	-11.51	2.06E-12	3.05E-08
ENST00000382689	DEFA1B	-11.86	2.51E-12	3.18E-08
ENST00000274063	SFRP2	3.25	2.35E-11	2.60E-07
ENST00000321429	DUOX1	-2.86	6.31E-11	6.22E-07
ENST00000345136	PLEC	-10.39	1.25E-10	1.11E-06
TCONS_00155523	-	-8.37	2.52E-10	2.03E-06
ENST00000368738	S100A9	-2.56	5.31E-10	3.93E-06
TCONS_00156753	ANKRD34B	-7.53	1.78E-09	1.21E-05
ENST00000360428	DSC3	6.59	2.45E-09	1.55E-05
ENST00000612277	HLCS	-8.49	2.68E-09	1.58E-05
ENST00000260227	MMP7	4.32	6.22E-09	3.45E-05
ENST00000566503	CYP1A1	-10.71	1.05E-08	5.49E-05
ENST00000271651	CTSK	2.43	1.12E-08	5.52E-05
ENST00000618265	CD177	-3.02	1.24E-08	5.77E-05
ENST00000257189	DSG3	8.58	1.56E-08	6.90E-05
ENST00000327857	DEFA3	-4.72	1.71E-08	7.23E-05
ENST00000297267	FNDC1	8.87	2.42E-08	9.76E-05
ENST00000376795	DNAJC3	-8.29	3.79E-08	1.46E-04
ENST00000290271	STC1	-2.75	5.38E-08	1.99E-04
TCONS_00199075	CAMSAP1	-7.02	8.57E-08	2.93E-04
ENST00000260302	MMP13	6.12	8.59E-08	2.93E-04
ENST00000305139	UGT1A6	5.73	9.46E-08	3.00E-04
ENST00000438257	DIO2	3.39	9.75E-08	3.00E-04
ENST00000336596	EPHA3	2.52	1.01E-07	3.00E-04
TCONS_00011236	CSF3R	-1.91	1.04E-07	3.00E-04
ENST00000407050	PPFIA2	6.33	1.05E-07	3.00E-04
ENST00000402114	STON1-GTF2A1L	10.10	1.27E-07	3.52E-04
ENST00000368737	S100A12	-4.11	1.40E-07	3.76E-04
ENST00000326245	ITLN1	7.77	1.53E-07	3.96E-04
ENST00000486742	LILRA5	-9.09	1.56E-07	3.96E-04
ENST00000360863	RGS22	10.22	1.70E-07	4.18E-04
TCONS_00181099	DGKI	9.06	1.96E-07	4.69E-04
ENST00000375544	ASPN	2.88	2.03E-07	4.74E-04
ENST00000373103	CSF3R	-3.16	2.13E-07	4.83E-04
ENST00000343975	HMGCR	-2.40	2.27E-07	5.04E-04
ENST00000544465	MAP7	-9.67	2.49E-07	5.39E-04
ENST00000372811	MFSD2A	-2.16	3.22E-07	6.79E-04
ENST00000303749	SDR16C5	-2.20	3.49E-07	7.18E-04

ENST00000369000	TACC2	-4.09	3.57E-07	7.18E-04
ENST00000248076	F2RL3	-4.01	3.64E-07	7.18E-04
TCONS_00092864	IGFL2	11.08	3.77E-07	7.26E-04
ENST00000357055	SLC30A6	-8.70	4.56E-07	8.53E-04
TCONS_00047434	CKAP4	-9.59	4.61E-07	8.53E-04
TCONS_00146692	-	3.87	4.98E-07	9.01E-04
ENST00000560721	ZEB1	-9.21	5.12E-07	9.08E-04
TCONS_00106912	ARMC9	8.59	5.81E-07	9.91E-04
ENST00000402687	SULF1	2.59	5.82E-07	9.91E-04
ENST00000217233	TRIB3	-2.52	6.01E-07	9.91E-04
ENST00000370552	HPSE2	4.32	6.04E-07	9.91E-04
ENST00000375840	STRADA	10.27	6.72E-07	1.07E-03
TCONS_00014606	NBPF10	-7.61	6.74E-07	1.07E-03
ENST00000635197	FASN	-2.81	7.59E-07	1.18E-03
ENST00000304625	RNASE2	-3.23	7.73E-07	1.18E-03
ENST00000382771	SERPINB5	7.10	8.23E-07	1.24E-03
ENST00000344604	ECM2	2.75	8.68E-07	1.28E-03
ENST00000618855	TMPRSS4	9.34	8.94E-07	1.30E-03
TCONS_00118140	PCED1A	-8.93	1.01E-06	1.44E-03
ENST00000508233	SPP1	3.78	1.06E-06	1.49E-03
ENST00000511884	PCDH7	2.73	1.07E-06	1.49E-03
ENST00000237623	SPP1	4.39	1.19E-06	1.63E-03
TCONS_00088862	CD226	-8.40	1.25E-06	1.67E-03
ENST00000514539	TACC2	-2.44	1.26E-06	1.67E-03
ENST00000443509	PRUNE2	2.39	1.30E-06	1.70E-03
ENST00000379749	POSTN	3.10	1.45E-06	1.86E-03
ENST00000452625	MYH11	3.01	1.52E-06	1.93E-03
ENST00000353609	FOSB	13.01	1.57E-06	1.96E-03
ENST00000345382	BNC1	6.24	1.71E-06	2.11E-03
ENST00000376618	CPT1A	-9.08	1.75E-06	2.13E-03
TCONS_00134506	Znf385b	3.12	1.80E-06	2.15E-03
ENST00000167586	KRT14	8.18	1.90E-06	2.24E-03
ENST00000272895	ABCA12	5.35	2.33E-06	2.72E-03
ENST00000297848	COL14A1	2.16	2.45E-06	2.83E-03
TCONS_00102770	-	-2.75	2.60E-06	2.94E-03
TCONS_00105486	SLC39A10	-7.99	2.62E-06	2.94E-03
TCONS_00026924	ZNF143	-10.10	2.74E-06	3.04E-03
TCONS_00023124	Yme1l1	-8.12	2.78E-06	3.04E-03
TCONS_00169664	COL10A1	5.40	3.08E-06	3.33E-03
TCONS_00167840	PTCHD4	4.40	3.21E-06	3.43E-03
ENST00000382692	DEFA1	-3.48	3.30E-06	3.49E-03
ENST00000375561	OGN	2.68	3.48E-06	3.63E-03
ENST00000280612	SLC7A11	-2.34	3.78E-06	3.90E-03
TCONS_00197751	C16orf89	-4.41	3.95E-06	4.02E-03
ENST00000306749	FASN	-2.74	4.08E-06	4.11E-03

ENST00000494014	EPHA3	3.13	4.18E-06	4.17E-03
ENST00000606794	APLNR	3.03	4.40E-06	4.34E-03
ENST00000620985	SAMD9	-7.58	4.87E-06	4.74E-03
TCONS_00162591	PHF3	9.26	4.97E-06	4.79E-03
ENST00000369236	GFRA1	2.02	5.11E-06	4.85E-03
TCONS_00069401	RSPRY1	10.94	5.15E-06	4.85E-03
TCONS_00070913	SPIRE2	8.75	5.88E-06	5.49E-03
ENST00000395080	SPP1	3.81	6.23E-06	5.70E-03
ENST00000400888	CCR2	-6.47	6.26E-06	5.70E-03
ENST00000394943	LIMA1	-9.31	6.34E-06	5.70E-03
ENST00000378702	DMD	-7.79	6.37E-06	5.70E-03
ENST00000380880	FREM1	2.07	6.55E-06	5.80E-03
TCONS_00174496	SDHAF3	-8.41	6.69E-06	5.88E-03
TCONS_00071349	ABCA3	-2.23	7.23E-06	6.25E-03
TCONS_00149876	-	-3.62	7.26E-06	6.25E-03
TCONS_00038688	-	4.43	7.68E-06	6.55E-03
ENST00000382892	NSD2	-6.97	7.88E-06	6.65E-03
TCONS_00161371	LY6G6F	-8.27	8.04E-06	6.73E-03
ENST00000016946	RGPD5	-9.39	8.42E-06	6.98E-03
ENST00000374512	PHF1	-10.15	8.62E-06	7.08E-03
ENST00000439203	DST	10.31	8.77E-06	7.13E-03
ENST00000302118	PCSK9	-2.56	8.88E-06	7.14E-03
TCONS_00147136	MAPK10	8.57	8.94E-06	7.14E-03
ENST00000326499	VNN2	-2.03	9.48E-06	7.50E-03
ENST00000624995	SLC27A3	-12.06	9.66E-06	7.58E-03
TCONS_00195574	AQP3	-10.79	9.82E-06	7.60E-03
TCONS_00041596	CFAP54	2.75	9.86E-06	7.60E-03
TCONS_00040556	PIP4K2C	-2.82	1.11E-05	8.46E-03
ENST00000393976	KRT15	5.07	1.12E-05	8.46E-03
ENST00000265753	EIF4H	-2.14	1.17E-05	8.77E-03
TCONS_00011996	USP24	10.71	1.18E-05	8.77E-03
TCONS_00162352	FAM83B	4.52	1.20E-05	8.79E-03
ENST00000219022	OLFM4	-4.43	1.20E-05	8.79E-03
TCONS_00041722	ANO4	6.43	1.24E-05	8.99E-03
TCONS_00121904	NRIP1	10.21	1.26E-05	9.11E-03
ENST00000458141	SULF1	2.63	1.28E-05	9.12E-03
ENST00000454381	GADL1	4.44	1.29E-05	9.18E-03
ENST00000393211	COL17A1	10.14	1.31E-05	9.19E-03
ENST00000238994	PPP1R3C	1.73	1.32E-05	9.24E-03
TCONS_00168553	-	-8.55	1.37E-05	9.45E-03
ENST00000353065	PROK2	-3.32	1.37E-05	9.45E-03
ENST00000457135	ARID2	-6.85	1.39E-05	9.48E-03
ENST00000379965	TRIM22	1.78	1.40E-05	9.48E-03
ENST00000512820	LIMCH1	10.50	1.46E-05	9.79E-03
ENST00000398165	CBS	-2.66	1.48E-05	9.85E-03

TCONS_00135192	ZNF852	-8.19	1.49E-05	9.89E-03
TCONS_00148171	GVQW1	3.02	1.51E-05	9.91E-03
ENST00000307142	SYNPO2	2.24	1.55E-05	1.01E-02
ENST00000433297	HMGCS1	-2.05	1.60E-05	1.04E-02
TCONS_00166867	BAG6	-5.66	1.62E-05	1.04E-02
TCONS_00108736	CDC42EP3	-9.68	1.63E-05	1.04E-02
TCONS_00045145	DHH	-7.29	1.70E-05	1.06E-02
ENST00000244869	EREG	4.37	1.71E-05	1.06E-02
ENST00000540367	CPT1A	8.85	1.72E-05	1.06E-02
ENST00000622074	GREM1	6.00	1.72E-05	1.06E-02
TCONS_00065772	CYP1A1	-9.38	1.73E-05	1.06E-02
TCONS_00171693	FOXK1	-8.67	1.74E-05	1.06E-02
ENST00000542664	HTR2A	3.27	1.75E-05	1.06E-02
ENST00000368222	CRABP2	3.10	1.82E-05	1.09E-02
TCONS_00017079	NUCKS1	1.96	1.82E-05	1.09E-02
TCONS_00162108	C6orf223	-2.64	1.83E-05	1.09E-02
ENST00000440293	HDAC7	-9.51	1.87E-05	1.10E-02
ENST00000599460	SYMPK	-8.21	1.93E-05	1.13E-02
ENST00000512158	CXCL14	2.98	1.98E-05	1.16E-02
ENST00000274532	TIMD4	4.29	2.03E-05	1.17E-02
TCONS_00065775	CYP1A1	-9.32	2.12E-05	1.22E-02
ENST00000370674	FHL1	2.94	2.15E-05	1.23E-02
ENST00000336577	MMP25	-2.94	2.17E-05	1.23E-02
ENST00000502486	BEND4	-5.82	2.18E-05	1.23E-02
ENST00000337571	CAPN3	6.94	2.31E-05	1.29E-02
ENST00000424424	PRNP	-10.14	2.35E-05	1.29E-02
ENST00000369538	AMPD1	3.52	2.37E-05	1.29E-02
ENST00000432233	LILRA5	-1.81	2.38E-05	1.29E-02
ENST00000381841	FGL1	-8.31	2.39E-05	1.29E-02
ENST00000560830	GREM1	4.07	2.40E-05	1.29E-02
ENST00000392027	ALPP	-3.16	2.41E-05	1.29E-02
ENST00000259407	BAAT	3.28	2.41E-05	1.29E-02
ENST00000340368	INSIG1	-1.50	2.42E-05	1.29E-02
ENST00000539068	FKBP5	-8.69	2.44E-05	1.29E-02
ENST00000397146	ITIH5	1.80	2.44E-05	1.29E-02
ENST00000265896	SQLE	-1.55	2.46E-05	1.29E-02
ENST00000305991	SLN	5.11	2.50E-05	1.31E-02
ENST00000325110	HMGCS1	-1.68	2.58E-05	1.34E-02
ENST00000543579	TCF12	2.57	2.61E-05	1.34E-02
TCONS_00050910	FLT3	-7.54	2.67E-05	1.36E-02
ENST00000334047	F3	-1.74	2.69E-05	1.36E-02
ENST00000344256	CDC25B	-1.66	2.70E-05	1.36E-02
TCONS_00154992	LPCAT1	-8.82	2.70E-05	1.36E-02
ENST00000440835	PTPRR	7.49	2.80E-05	1.39E-02
ENST00000271588	HMCN1	1.44	2.82E-05	1.39E-02

ENST00000402914	MACROD2	-2.45	2.84E-05	1.39E-02
TCONS_00059208	NRDE2	-6.76	2.85E-05	1.39E-02
ENST00000400286	SLITRK6	2.07	2.86E-05	1.39E-02
TCONS_00033345	DCDC5	7.36	2.88E-05	1.39E-02
TCONS_00158866	PDGFRB	-10.76	2.89E-05	1.39E-02
TCONS_00056205	TECPR2	9.00	2.89E-05	1.39E-02
TCONS_00177625	OSBPL3	9.46	2.96E-05	1.42E-02
TCONS_00007505	C16orf89	2.58	2.97E-05	1.42E-02
TCONS_00165171	FNDC1	4.26	3.09E-05	1.46E-02
ENST00000477922	MGAM2	-3.39	3.12E-05	1.46E-02
ENST00000251822	RHOBTB2	-1.61	3.15E-05	1.46E-02
ENST00000248484	TNFRSF19	1.84	3.15E-05	1.46E-02
TCONS_00115996	LAMP5	5.03	3.16E-05	1.46E-02
ENST00000458406	SLC12A6	-7.41	3.16E-05	1.46E-02
ENST00000243924	PI3	-2.73	3.18E-05	1.46E-02
ENST00000221847	EBI3	-10.24	3.25E-05	1.48E-02
TCONS_00056178	DYNC1H1	-6.78	3.29E-05	1.49E-02
ENST00000356462	NEDD4L	-7.68	3.29E-05	1.49E-02
ENST00000226230	TMEM97	-1.72	3.30E-05	1.49E-02
ENST00000350997	FADS1	-1.96	3.45E-05	1.55E-02
ENST00000347624	LRRC15	3.31	3.55E-05	1.58E-02
ENST00000252242	KRT5	4.98	3.56E-05	1.58E-02
TCONS_00092228	SHKBP1	-7.23	3.57E-05	1.58E-02
ENST00000360814	GLT8D2	2.14	3.66E-05	1.61E-02
TCONS_00075657	NTN1	10.84	3.70E-05	1.62E-02
ENST00000513317	ACSL1	-1.87	3.76E-05	1.63E-02
TCONS_00198147	INIP	-8.18	3.82E-05	1.65E-02
ENST00000345517	ACTG2	2.37	3.95E-05	1.70E-02
ENST00000404525	LTBP1	1.72	3.96E-05	1.70E-02
ENST00000640036	SCN1A	-6.26	4.06E-05	1.73E-02
ENST00000404917	IL1RL1	10.31	4.22E-05	1.78E-02
TCONS_00132581	ZNF91	2.11	4.22E-05	1.78E-02
ENST00000330753	FLRT2	1.73	4.29E-05	1.80E-02
ENST00000370571	COL24A1	2.36	4.30E-05	1.80E-02
ENST00000535035	DHCR24	-10.80	4.48E-05	1.87E-02
ENST00000495027	DNAH12	2.75	4.58E-05	1.90E-02
ENST00000367750	FMO1	-8.15	4.64E-05	1.90E-02
ENST00000429713	SYNPO2	1.86	4.66E-05	1.90E-02
ENST00000215727	SERPIND1	3.49	4.67E-05	1.90E-02
ENST00000296326	ZDHHC19	-4.40	4.69E-05	1.90E-02
ENST00000402881	MIEF1	-8.20	4.70E-05	1.90E-02
ENST00000391807	KLK7	8.01	4.83E-05	1.95E-02
ENST00000357525	PLN	2.40	4.90E-05	1.97E-02
ENST00000423302	MRVI1	2.10	4.93E-05	1.97E-02
TCONS_00167841	PTCHD4	5.91	5.00E-05	1.99E-02

TCONS_00071544	ZNF597	-7.78	5.14E-05	2.03E-02
ENST00000311208	KRT17	4.03	5.16E-05	2.03E-02
ENST00000296575	HHIP	-2.12	5.22E-05	2.05E-02
TCONS_00103810	MGAT5	10.52	5.33E-05	2.08E-02
ENST00000270879	FCN3	-2.58	5.42E-05	2.10E-02
TCONS_00039066	SSPN	2.30	5.43E-05	2.10E-02
ENST00000373106	CSF3R	-1.66	5.58E-05	2.14E-02
TCONS_00139190	ZMAT3	1.59	5.59E-05	2.14E-02
ENST00000342743	TMEM215	5.33	5.60E-05	2.14E-02
TCONS_00102328	-	2.92	5.64E-05	2.15E-02
ENST00000554719	NRXN3	7.79	5.72E-05	2.17E-02
ENST00000368498	GOPC	3.14	5.79E-05	2.18E-02
ENST00000451104	PITRM1	-6.14	5.81E-05	2.18E-02
ENST00000417640	NECAB1	-2.24	5.89E-05	2.20E-02
ENST00000252444	LDLR	-1.50	5.92E-05	2.20E-02
ENST00000355527	DHCR7	-2.00	5.93E-05	2.20E-02
ENST00000398738	PTCHD4	3.52	5.97E-05	2.20E-02
TCONS_00048377	-	-2.95	6.01E-05	2.20E-02
ENST00000367485	PRG4	2.19	6.02E-05	2.20E-02
ENST00000309649	RNF146	-7.77	6.02E-05	2.20E-02
TCONS_00134231	-	3.73	6.13E-05	2.23E-02
TCONS_00016951	CHIT1	3.34	6.15E-05	2.23E-02
ENST00000419673	ARHGAP28	-8.88	6.21E-05	2.24E-02
ENST00000293599	AQP5	2.24	6.34E-05	2.28E-02
ENST00000509507	CALCOCO2	-7.46	6.51E-05	2.33E-02
TCONS_00015544	OR6K3	-7.00	6.65E-05	2.37E-02
ENST00000338682	ANKRD34B	-4.81	6.74E-05	2.38E-02
TCONS_00149003	DDX60L	-7.61	6.74E-05	2.38E-02
ENST00000377693	IGFL2	10.07	6.77E-05	2.38E-02
ENST00000396721	SDR16C5	-1.76	6.82E-05	2.39E-02
ENST00000630130	CFH	1.79	6.95E-05	2.42E-02
ENST00000260843	GPR87	5.36	6.97E-05	2.42E-02
TCONS_00051440	-	3.07	7.04E-05	2.44E-02
ENST00000425538	MYOCD	2.03	7.10E-05	2.45E-02
TCONS_00201559	DIAPH2	-5.15	7.38E-05	2.53E-02
ENST00000430069	TBL1XR1	1.82	7.38E-05	2.53E-02
ENST00000367048	ACAT2	-1.58	7.48E-05	2.55E-02
ENST00000376238	SLC28A3	2.91	7.59E-05	2.58E-02
ENST00000358399	GDA	6.33	7.75E-05	2.62E-02
ENST00000345633	CASP7	-7.70	7.84E-05	2.63E-02
ENST00000296027	CXCL5	2.53	7.84E-05	2.63E-02
ENST00000264381	BCHE	1.97	7.86E-05	2.63E-02
ENST00000312428	DNAH7	2.48	7.97E-05	2.65E-02
TCONS_00178406	NUDCD3	-7.23	7.97E-05	2.65E-02
ENST00000455783	TMEM229A	4.82	8.11E-05	2.68E-02

ENST00000291759	LILRA4	6.59	8.14E-05	2.68E-02
ENST00000396934	BTN3A2	10.61	8.23E-05	2.70E-02
ENST00000373069	SLC25A25	-7.34	8.41E-05	2.75E-02
ENST00000225275	MPO	-3.27	8.44E-05	2.75E-02
ENST00000369409	PHGDH	-1.80	8.51E-05	2.76E-02
ENST00000376180	ITGBL1	1.95	8.52E-05	2.76E-02
ENST00000396396	PGM5	1.54	8.64E-05	2.79E-02
ENST00000254090	FMO5	-1.92	8.76E-05	2.81E-02
TCONS_00176170	ARHGEF5	5.39	8.80E-05	2.82E-02
ENST00000525171	NAALAD2	7.97	8.94E-05	2.85E-02
ENST00000327883	ADAMTSL1	4.71	9.14E-05	2.89E-02
TCONS_00124459	MICALL1	-8.80	9.14E-05	2.89E-02
ENST00000371022	WDR78	9.32	9.17E-05	2.89E-02
TCONS_00144325	-	3.76	9.20E-05	2.89E-02
ENST00000286758	CXCL13	3.87	9.27E-05	2.90E-02
ENST00000318876	ZFYVE1	-7.79	9.29E-05	2.90E-02
ENST00000359576	CLASP2	-8.28	9.54E-05	2.96E-02
TCONS_00042145	FAM222A	-3.27	9.54E-05	2.96E-02
ENST00000261973	RBM25	-8.20	9.66E-05	2.99E-02
ENST00000300113	CHP2	8.85	9.81E-05	3.02E-02
TCONS_00132673	PIK3CA	-6.71	9.83E-05	3.02E-02
TCONS_00071513	MEFV	-1.91	9.87E-05	3.02E-02
ENST00000381174	XG	5.74	1.00E-04	3.05E-02
ENST00000377991	TMEM8B	-7.35	1.02E-04	3.10E-02
TCONS_00038715	-	6.45	1.03E-04	3.10E-02
TCONS_00197291	SEMA4D	5.30	1.03E-04	3.10E-02
ENST00000519678	ASPH	-2.93	1.04E-04	3.12E-02
ENST00000296464	HSPA4L	2.17	1.04E-04	3.12E-02
ENST00000408995	FHL2	3.11	1.05E-04	3.12E-02
TCONS_00008145	CR2	9.50	1.12E-04	3.33E-02
ENST00000217957	VSIG1	4.36	1.12E-04	3.33E-02
ENST00000455444	TLE2	-7.67	1.15E-04	3.41E-02
ENST00000558322	DUOX1	-2.81	1.18E-04	3.46E-02
ENST00000558518	LDLR	-1.65	1.18E-04	3.47E-02
ENST00000409968	THSD7B	5.77	1.18E-04	3.47E-02
ENST00000317716	CDHR3	2.98	1.19E-04	3.47E-02
TCONS_00068825	SRCAP	-6.16	1.21E-04	3.52E-02
TCONS_00116136	MACROD2	-2.09	1.22E-04	3.54E-02
TCONS_00129576	ROBO2	2.08	1.22E-04	3.52E-02
ENST00000389394	DNAH6	2.38	1.23E-04	3.54E-02
ENST00000262941	ZSCAN25	-8.31	1.23E-04	3.54E-02
ENST00000409762	DYSF	-8.27	1.25E-04	3.57E-02
ENST00000375099	UBXN10	2.23	1.25E-04	3.57E-02
ENST00000427120	ABCC5	1.80	1.27E-04	3.58E-02
TCONS_00165789	TXNDC5	-4.34	1.27E-04	3.58E-02

ENST00000357077	ANK2	2.16	1.28E-04	3.58E-02
ENST00000393481	TES	-10.49	1.28E-04	3.58E-02
ENST00000489843	ARF4	-8.34	1.29E-04	3.58E-02
ENST00000341552	CFAP65	8.51	1.29E-04	3.58E-02
TCONS_00114715	CXCR2	-2.69	1.29E-04	3.58E-02
ENST00000328224	KCNA4	3.08	1.29E-04	3.58E-02
ENST00000301730	RNPS1	-10.50	1.29E-04	3.58E-02
TCONS_00062685	ARNT2	-8.31	1.30E-04	3.58E-02
ENST00000249749	DLL4	-1.88	1.31E-04	3.60E-02
TCONS_00047895	VSIG10	-1.81	1.32E-04	3.62E-02
ENST00000418500	COL10A1	10.00	1.34E-04	3.66E-02
ENST00000562522	ITGAX	-9.36	1.34E-04	3.66E-02
ENST00000619049	ZMYND8	5.35	1.39E-04	3.77E-02
ENST00000592452	ZNF532	9.13	1.39E-04	3.77E-02
ENST00000396097	LPIN1	9.68	1.40E-04	3.77E-02
ENST00000219596	MEFV	-2.16	1.40E-04	3.77E-02
ENST00000239374	CCDC170	2.18	1.41E-04	3.77E-02
TCONS_00017001	PIK3C2B	-6.89	1.41E-04	3.77E-02
TCONS_00071844	-	6.77	1.42E-04	3.80E-02
TCONS_00163041	SLC35A1	-7.92	1.43E-04	3.80E-02
TCONS_00156754	ANKRD34B	-4.49	1.45E-04	3.84E-02
ENST00000399202	FAM214A	-8.12	1.45E-04	3.84E-02
ENST00000304749	CST1	11.06	1.47E-04	3.86E-02
ENST00000619531	HHLA2	3.70	1.47E-04	3.86E-02
ENST00000393567	HYDIN	2.58	1.49E-04	3.91E-02
ENST00000613909	ITIH5	1.79	1.50E-04	3.92E-02
TCONS_00037178	TRIM29	3.20	1.51E-04	3.94E-02
TCONS_00148009	NDNF	-7.66	1.55E-04	4.03E-02
TCONS_00175566	FLNC	-8.75	1.56E-04	4.04E-02
ENST00000444490	ECM2	3.10	1.57E-04	4.05E-02
TCONS_00204285	TMEM78	7.27	1.57E-04	4.05E-02
ENST00000623662	ABC7	-1.71	1.61E-04	4.14E-02
ENST00000456764	IGFBP2	1.84	1.62E-04	4.15E-02
ENST00000295417	FZD5	-1.30	1.63E-04	4.18E-02
ENST00000614163	CYP4B1	11.58	1.65E-04	4.20E-02
TCONS_00093709	MYADM	-8.08	1.65E-04	4.20E-02
TCONS_00035379	ARAP1	8.68	1.67E-04	4.22E-02
ENST00000334025	RGS7BP	1.98	1.67E-04	4.22E-02
ENST00000393054	ATP6V0A4	8.48	1.72E-04	4.30E-02
ENST00000548409	KRT5	6.88	1.72E-04	4.30E-02
ENST00000409809	COL6A3	-8.73	1.73E-04	4.30E-02
TCONS_00004366	CSF1	-7.88	1.73E-04	4.30E-02
ENST00000522671	SDR16C5	-2.92	1.73E-04	4.30E-02
ENST00000393957	TPPP3	1.97	1.73E-04	4.30E-02
ENST00000429102	CDH3	2.37	1.74E-04	4.30E-02

ENST00000487947	PDCD10	-8.11	1.74E-04	4.30E-02
ENST00000308191	PDE7B	2.40	1.75E-04	4.31E-02
ENST00000592436	FOSB	12.53	1.78E-04	4.36E-02
ENST00000303395	SCN1A	-2.18	1.80E-04	4.41E-02
ENST00000418488	IL5RA	2.32	1.81E-04	4.42E-02
TCONS_00181020	PLXNA4	-7.00	1.84E-04	4.49E-02
ENST00000540876	CLDN2	3.96	1.85E-04	4.50E-02
ENST00000634418	RYR3	3.72	1.87E-04	4.53E-02
ENST00000482026	UGT1A3	8.77	1.90E-04	4.59E-02
TCONS_00187511	ARFGEF1	-7.82	1.91E-04	4.61E-02
TCONS_00113762	NEMP2	8.46	1.93E-04	4.64E-02
ENST00000318160	GREM2	2.66	1.95E-04	4.66E-02
TCONS_00124268	HMGXB4	5.64	1.96E-04	4.67E-02
ENST00000323523	TMEM45A	1.92	1.96E-04	4.67E-02
ENST00000431818	IFT122	-6.32	1.97E-04	4.68E-02
ENST00000295213	SPATA18	2.14	1.97E-04	4.67E-02
ENST00000353479	COL17A1	4.82	2.00E-04	4.73E-02
TCONS_00007294	HMCN1	1.30	2.01E-04	4.75E-02
ENST00000381404	ADGRE1	-6.93	2.04E-04	4.79E-02
ENST00000551035	OS9	-5.86	2.05E-04	4.81E-02
TCONS_00010071	FBXO2	-8.88	2.10E-04	4.91E-02
ENST00000261507	MSMO1	-1.47	2.13E-04	4.97E-02

Abbreviations: FC, Fold-Change in IPF compared to HC; “TCONS_”, new transcript.

Table S5. The main GO terms, KEGG pathways and DETs enriched in those.

Important BPs of GO, and KEGG pathways	Enriched DETs
Extracellular matrix organization	SPP1, MMP7, MMP13, CTSK, SFRP2, COL14A1, FLRT2, ECM2, HPSE2, SERPINB5, KLK7, SPP1, SULF1, MYH11, SULF1, GREM1, GREM1
Extracellular structure organization	DMD, SPP1, MMP7, MMP13, CTSK, SFRP2, COL14A1, FLRT2, ECM2, HPSE2, SERPINB5, KLK7, SPP1, SULF1, MYH11, SULF1, GREM1, GREM1, COL10A1
Small molecule metabolic process	TRIB3, MPO, MSMO1, SQLE, PCSK9, SDR16C5, FASN, HMGCS1, VNN2, INSIG1, HMGCR, FADS1, DHCR7, ACAT2, FMO1, PHGD, SLC25A25_, CPT1A, CYP1A1, SDR16C5, CBS, CCR2, MACROD2, HMGCS1, ACSL1, LDLR_, HLCS, SLC27A3, DYNC1H1_, CYP1A1, CYP1A1, ABCA3, MACROD2, DDX60L, LPCAT1, SDHAF3
Lipid metabolic process	TRIB3, MSMO1, SQLE, PCSK9, SDR16C5, FASN, HMGCS1, INSIG1, HMGCR, FADS1, DHCR7, ACAT2, MFSD2A, CPT1A, CYP1A1, SDR16C5, HMGCS1, ACSL1_, LDLR, SLC27A3, PIP4K2C, DHH, CYP1A1, CYP1A1, LPCAT1, PDGFRB
Ecm-receptor interaction	SPP1, SPP1,SPP1
Toll-like receptor signaling pathway	SPP1, CTSK, SPP1, SPP1
Cytokine-cytokine receptor interaction	TNFRSF19, CXCL13, CXCL5, IL5RA, CXCL14
Fatty acid metabolism	FASN, FADS1, ACAT2, CPT1A, ACSL1, FASN
Fatty acid biosynthesis	FASN, ACSL1, FASN
Cytokine-cytokine receptor interaction	CSF1, CSF3R, FLT3, CPT1A, PDGFRB, CSF3R, CSF3R, CCR2

Some BP term or KEGG pathways have one or more transcripts, presenting the appearance of multiple same genes in this table. Abbreviations. DETs, Differentially Expressed Transcripts; BP, Biological Process.

SUPPLEMENTARY FIGURE LEGENDS

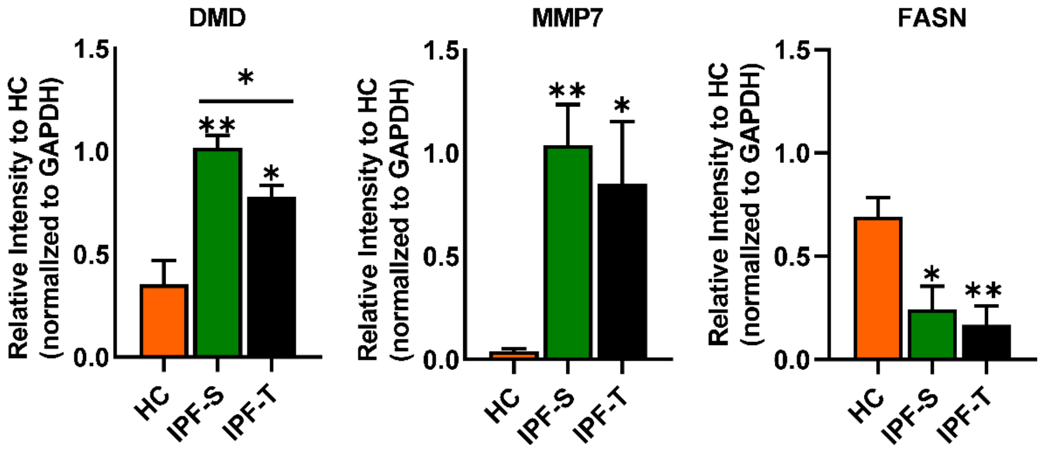

Figure S1. The protein expression of DMD, MMP7, and FASN at different stages of IPF and HC, as measured by western blot. Each group had 4 samples. And the differences between HC vs. IPF-S, HC vs. IPF-T, and IPF-S vs. IPF-T are considered significant at: **P < 0.01; *P<0.05; and no *,not significant. Abbreviation. HC, healthy controls; IPF-S, IPF samples with early-stage from surgical lung biopsy; IPF-T, IPF samples with transplant-stage from lung transplantation.

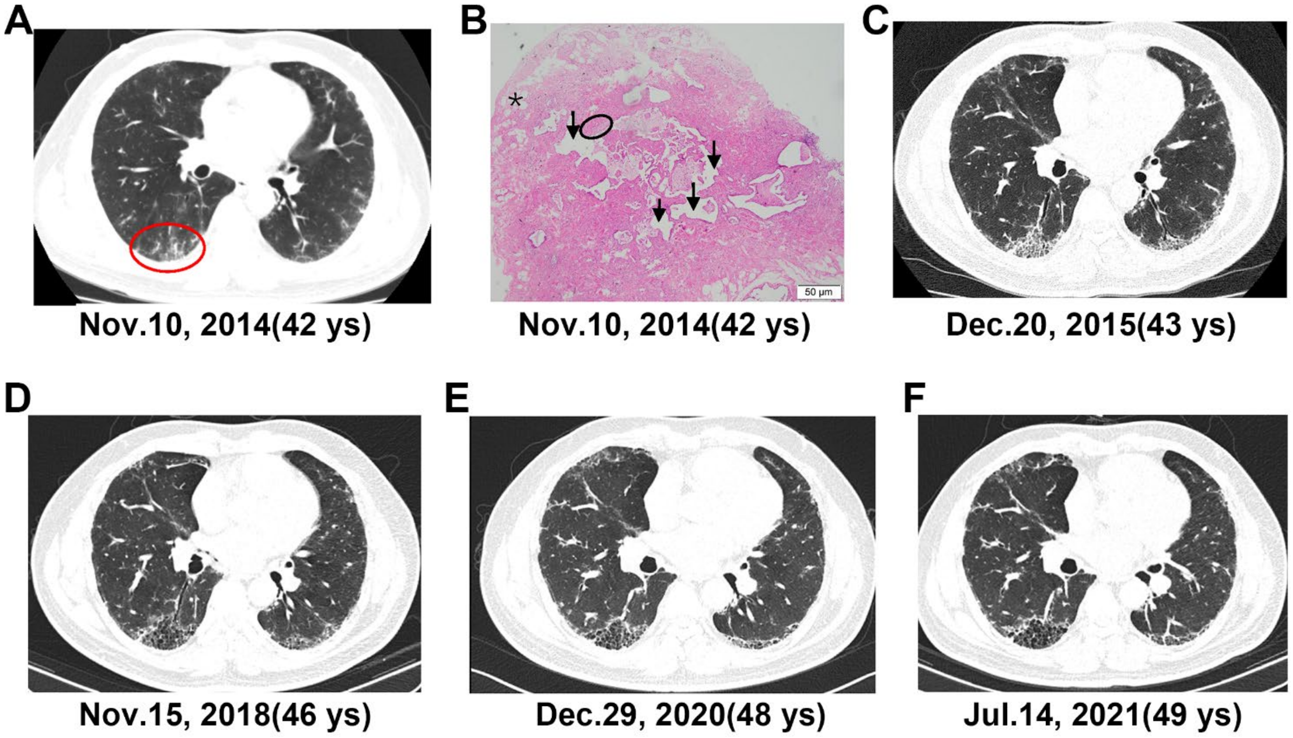

Figure S2 The HRCT topographic images of the youngest patient following up from 42 to 49 years and the pathological image at the time of diagnosis at age 42. A shows a HRCT image, representing "indeterminate UIP" characterized by subpleural, basal-predominant (often heterogeneous) subtle reticulation, mild GGO or distortion. The SLB area was highlighted (red circle marked). B: a histopathology patterns of UIP, characterized by dense fibrosis with a predilection for subpleural and paraseptal parenchyma with associated architectural distortion in the form of microscopic honeycomb change (black arrows pointed) juxtaposed with relatively unaffected lung parenchyma (*), patchy involvement of lung parenchyma by fibrosis, and fibroblast foci (black circle). C: "probable UIP" characterized by reticular pattern with bronchiolectasis. D-F: a typical UIP with characteristics of subpleural, basal-predominant (heterogeneous) honeycombing and bronchiolectasis.
