# Supplementary material for: Complex Involvement of the Extracellular Matrix, Immune Effect, and Lipid Metabolism in the Development of Idiopathic Pulmonary Fibrosis
Source: Front Mol Biosci. 2022 Jan 31;8:800747. doi: 10.3389/fmolb.2021.800747 (PMC8841329; doi:10.3389/fmolb.2021.800747)
Supplement: Supplementary file 2 [file DataSheet1.PDF]

广州医科大学附属第一医院科研项目审查伦理委员会

临床试验会议审批件

医研伦审 2020 第 71 号

<table>
<tr><td>项目名称</td><td colspan="5">基于转录组学的间质性肺疾病致病机制研究</td></tr>
<tr><td>申办者</td><td colspan="2">广州医科大学附属第一医院呼吸内科</td><td colspan="2">项目负责人</td><td>罗群</td></tr>
<tr><td>审查类别</td><td colspan="5">会议审查（重审）</td></tr>
<tr><td rowspan="3">表决结果</td><td>委员人数 15 人</td><td>出席人数 12 人</td><td colspan="2">回避人数 0 人</td><td>弃权人数 0 人</td></tr>
<tr><td>同意</td><td>作必要的修正后同意</td><td>作必要的修正后重审</td><td>不同意</td><td>终止或暂停先前批准的试验</td></tr>
<tr><td>12</td><td>0</td><td>0</td><td>0</td><td>0</td></tr>
<tr><td>结论</td><td colspan="5">同意</td></tr>
</table>

结合此前对该临床研究的伦理审查意见，现已对申办方提交的材料进行补充备案，同意批准“基于转录组学的间质性肺疾病致病机制研究”的实施。

伦理委员会主任委员签字：[illegible] 日期：2020-3-20

广州医科大学附属第一医院医学伦理委员会（盖章）

（印章：广州医科大学附属第一医院医学伦理委员会）

备注	1. 修改后同意/重审项目，应将修改后文件及时反馈伦理委员会，进行进一步审查。<br>2. 不同意/终止或暂停项目，批件发出 2 周内可向伦理委员会就有关事项做出解释或提出申诉。<br>3. 临床试验应严格按照本伦理委员会批准的文件执行。在试验实施过程中，如对试验方案、知情同意书等文件做任何修改，应及时向本伦理委员会提交变更申请，补充更新文件，经伦理委员会重新审查批准后，方可执行。<br>4. 发生严重不良事件及可能影响风险受益的任何事件和新信息须及时报告本伦理委员会。<br>5. 定期/年度跟踪审查项目，于到期后 1 周内提交试验进度情况报告。如有不依从/违背方案或暂停/提前终止的试验项目，应及时以书面文件告知本伦理委员会。试验结束后，须及时向伦理委员会提交结题报告。<br>6. 本批件有效期为 1 年（自批准之日起）。若在有效期内未启动项目，则本批件自动终止。<br>7. 本委员会依据 GCP 和国家法规以及 ICH-GCP 的要求操作。

审查日期：2020-03-13	审查地点：广州医科大学附属第一医院交运院区 1301 会议室	
联系方式：广州市越秀区沿江西路 151 号（邮编 510120）		
电话：020-83062938	传真：020-83389471	联系人：余达加

广州医科大学附属第一医院科研项目审查伦理委员会

临床试验会议审批件（目录页）

医研伦审 **2020** 第 **71** 号

项目审查通过的最终资料目录及版本号：

序号	文件名	版本号	日期
1.伦理递交函	1.1 复审申请表	NA	2020 年 03 月 10 日
	1.2 科研伦理审查申请表	NA	2020 年 03 月 10 日
2.方案	临床试验方案	V2.0	2020 年 02 月 20 日
3.知情同意书	知情同意书	V2.0	2020 年 02 月 20 日
4.简历	研究者简历（罗群）	NA	NA
5.其他	5.1 临床研究技术服务合同初稿	NA	NA
	5.2 公司简介（广州基迪奥生物科技有限公司）	NA	NA
	5.3 营业执照（广州基迪奥生物科技有限公司）	91440113589539753B	2017 年 05 月 26 日

审查日期：2020-03-13

广州医科大学附属第一医院科研项目审查伦理委员会

临床试验会议审批件（专家签到页）

审查日期：2020 年 3 月 13 日 上午 09:00

审查地点：交运院区 1301 会议室

出席名单	性别	单位名称	职务/职称	专业	伦理委员会职务	出席签名
徐评议	男	广州医科大学附属第一医院	神经内科主任/教授	内科	主任委员	[illegible]
周玉民	男	广州医科大学附属第一医院	呼吸内科主任/教授	呼吸内科	副主任委员	[illegible]
王薇	女	广州医科大学附属第一医院	妇产科主任/教授	医院管理	副主任委员	[illegible]
高兴成	男	广州医科大学附属第一医院	科教副院长/教授	泌尿外科	委员	[illegible]
李立	女	广东广信君达律师事务所	初级律师	法律	委员	[illegible]
洪巧妍	女	广州市越秀区人民街道办事处计生办（退休）	科长	管理	委员	[illegible]
刘荣	男	广州医科大学附属第一医院	急诊科/副主任医师	急诊	委员	[illegible]
吴文起	男	广州医科大学附属第一医院	泌尿外科实验室副主任/教授	外科	委员	[illegible]
高国贞	女	广州医科大学附属第一医院	护理部副主任/主任护师	护理	委员	[illegible]
欧阳斌	女	广州医科大学附属第一医院	医务科副科长/主任医师	外科	委员	
魏理	女	广州医科大学附属第一医院	药学部主任/主任药师	药学	委员	[illegible]
谭志坚	男	广州医科大学附属第一医院	设备科副科长/高级工程师	医疗器械	委员	
陈小清	男	广州医科大学附属第一医院	科研科科长/主治医师	中医	委员	[illegible]
王欣璐	女	广州医科大学附属第一医院	核医学科主任/主任医师	核医学科	委员	[illegible]
韩茜	女	广州医科大学附属第一医院	呼吸内科副主任/副主任医师	呼吸内科	委员	
委员人数 **15** 人			实到委员人数 12 人			
备注：						

广州医科大学附属第一医院医学伦理委员会

2020 年 3 月 13 日

（地址：广州市越秀区沿江西路 151 号 电话：020-83062938 邮编：510120）
